# Lymphatic Filariasis in Mainland Southeast Asia: A Systematic Review and Meta-Analysis of Prevalence and Disease Burden

**DOI:** 10.3390/tropicalmed2030032

**Published:** 2017-07-27

**Authors:** Benjamin F. R. Dickson, Patricia M. Graves, William J. McBride

**Affiliations:** 1 College of Medicine & Dentistry, James Cook University, Cairns, QLD 4870, Australia; 2 College of Public Health, Medical and Veterinary Sciences, James Cook University, Cairns, QLD 4870, Australia; patricia.graves@jcu.edu.au; 3 College of Medicine & Dentistry, James Cook University, Cairns, QLD 4870, Australia; john.mcbride@jcu.edu.au

**Keywords:** lymphatic filariasis, Southeast Asia, prevalence, infection, morbidity, lymphoedema, hydrocoele

## Abstract

Accurate prevalence data are essential for the elimination of lymphatic filariasis (LF) as a public health problem. Despite it bearing one of the highest burdens of disease globally, there remains limited reliable information on the current epidemiology of filariasis in mainland Southeast Asia. We conducted a systematic review and meta-analysis of available literature to assess the recent and current prevalence of infection and morbidity in the region. Fifty-seven journal articles and reports containing original prevalence data were identified, including over 512,010 participants. Data were summarised using percentage prevalence estimates and a subset combined using a random effects meta-analysis by country and year. Pooled estimates for microfilaraemia, immunochromatographic card positivity and combined morbidity were 2.64%, 4.48% and 1.34% respectively. Taking into account pooled country estimates, grey literature and the quality of available data, we conclude that Lao People's Democratic Republic (PDR), Myanmar and Northeast India demonstrate ongoing evidence of LF transmission that will require multiple further rounds of mass drug administration. Bangladesh, Malaysia, Thailand and Vietnam appear close to having eliminated LF, whilst Cambodia has already achieved elimination status. We estimate that the burden of morbidity is likely high in Thailand; moderate in Cambodia, Myanmar, and Northeast India; and low in Bangladesh. There was insufficient evidence to accurately estimate the disease burden in Lao PDR, Malaysia or Vietnam. The results of this study indicate that whilst considerable progress toward LF elimination has been made, there remains a significant filariasis burden in the region. The results of this study will assist policy makers to advocate and budget for future control programs.

## 1. Introduction

Lymphatic filariasis (LF) is a mosquito-borne tropical disease that affects 67.88 million people in 73 countries worldwide [1,2,3]. It is caused by infection with the nematodes *Wuchereria bancrofti (Wb)*, *Brugia malayi (Bm)* or *Brugia timori*. Chronic infection causes lymphatic dysfunction, resulting in severe morbidity from progressive, irreversible swelling of the limbs and genitals. LF is a significant cause of permanent disability worldwide, accounting for an estimated 19.43 million cases of hydrocoele, 16.68 million cases of lymphoedema and 2.02 million disability-adjusted life years lost [3,4].

In recognition of the significant worldwide burden of LF, the World Health Organization (WHO) established the Global Program to Eliminate LF (GPELF) calling for the elimination of LF by 2020 [1,5]. The program adopted a two-pronged approach: first, interrupt transmission through annual single-dose mass drug administration (MDA) of entire at-risk populations with albendazole in combination with either diethylcarbamazine (DEC) or ivermectin for a minimum of five years; and, second, alleviate the significant morbidity burden associated with the disease. 

Numerous methods are currently used to diagnose LF infection. Traditionally, thick blood smears (TBS) were used to identify microfilaraemia (mf) in the peripheral circulation. TBS have now been largely superseded by more sensitive antigen-based tests but are still used as a measure of potential infectivity and ongoing transmission. Antigen tests include immunochromatographic card tests (ICT) and Og4C3 enzyme-linked immunosorbent assays (ELISA) which use monoclonal antibodies to detect the excretory-secretory antigens produced by adult filarial worm infection [6,7]. Because they detect a different stage of the life-cycle and do not require adult worms to be producing microfilariae, they are two- to fivefold more sensitive than TBS [8]. Antibody-based tests detect circulating IgG4 antibodies against Bm14 antigen (*B. malayi* and *W. bancrofti)* or BmR1 (*B. malayi* only) [9]. Whilst highly sensitive and specific, antibody prevalence cannot prove current infection because antibodies remain elevated for many years after treatment [9]. Urinary antibody tests have been trialled but are less sensitive than blood-based antibody tests [8,10]. PCR assays to detect LF DNA in humans are also available, but are not used routinely because they require advanced laboratory facilities and are less sensitive than other methods [8,10].

LF is endemic in all countries within mainland Southeast Asia [1,2,5]. The geographical region spans both the WHO Southeast Asia and Western Pacific regions and includes Cambodia, Lao People’s Democratic Republic (PDR), Myanmar, Thailand, Vietnam and Malaysia. Emerging data suggest a possibly high prevalence of filariasis in Myanmar. Given the country’s significant shared border with Northeast India and Bangladesh, both were also included. 

The WHO Southeast Asia and Western Pacific regions account for 55.7% of the at-risk population, with 94.6% of reported lymphoedema cases and 85.2% of reported hydrocoele cases globally [2]. The areas considered here (mainland Southeast Asia plus Northeast India and Bangladesh) account for over a fifth of the population of these two regions. Elimination of LF in this area would therefore have a significant impact on the global disease burden. LF in these countries is caused by *W. Bancrofti* and *B. malayi*, and transmitted mainly by *Culex quinquefasciatus,* with some contribution by *Aedes* spp. and *Mansonia* spp. mosquitoes [11].

All countries in this review have commenced elimination programs. Elimination as a public health problem has been validated in Cambodia, whilst Bangladesh, Thailand and Vietnam have transitioned to post-MDA surveillance. The remaining countries are still conducting MDA [1,2,5]. 

WHO country validation of ‘elimination of LF as a public health problem’ requires a set of surveys and steps to determine whether the prevalence is below the target level (upper 95% CI of 2% in *Culex* spp. transmission areas such as Southeast Asia) [12]. One important milestone is the passing of three consecutive transmission assessment surveys (TAS) conducted on six- to seven-year-old children in each defined geographical evaluation unit (EU). The TAS survey sets critical cut-off values for the number of positive children that must not be exceeded for the EU to pass [13]. The sample sizes and critical cut-off values for the TAS in *Culex* spp. transmission areas are designed so that an EU has (1) at least a 75% chance of passing if the true prevalence of antigenaemia is 1.0% (half the target level); and (2) no more than a 5% chance of incorrectly passing if the true prevalence of antigenaemia is ≥2% [13]. 

Despite the significant LF burden in this region, there remain limited reliable data on the current prevalence of infection and morbidity. Whilst previous reviews have examined this topic, all are significantly out-dated or incomplete [14,15,16]. Reliable, current data on the prevalence of LF are required for the implementation and evaluation of elimination programs as well as future advocacy efforts. Accordingly, we conducted a systematic review and meta-analysis of publicly-available data in studies and grey literature to assess the recent (since the early 1990s) and current prevalence of LF infection and morbidity in mainland Southeast Asia, Bangladesh and Northeast India.

## 2. Materials and Methods

### 2.1. Protocol

This systematic review is reported using the Preferred Reporting Items for Systematic Reviews and Meta-Analysis (PRISMA) guidelines [17]. A review protocol was registered with PROSPERO international prospective register of systematic reviews, which can be viewed online [18].

### 2.2. Information Sources

Information was gathered through three sources: (1) a literature search of PubMed and MEDLINE databases; (2) a web-based search of the WHO library for relevant publications; and (3) direct contact with regional and national LF Program directors to obtain National LF Program reports [19]. Countries with National LF Programs submit annual reports to their respective WHO regional office outlining their MDA and surveying activities for the preceding year. A summary of the data is published annually in a regional WHO meeting report. Reference lists of all papers were screened to identify additional publications. 

### 2.3. Search Strategy

A literature search was conducted for available publications on PubMed and MEDLINE via OvidSP up until 11 December 2016 by one investigator. The search strategy used for both databases was: (lymphatic filariasis or Wuchereria bancrofti or Brugia timori or Brugia malayi or lymphoedema or hydrocoele or elephantiasis or microfilariae or microfilaraemia) AND ((Myanmar or Burma or Thailand or Laos or Cambodia or Vietnam or Malaysia or Bangladesh) OR (India and Assam or Meghalaya or Nagaland or Manipur or Tripura or Mizoram or Arunachal Pradesh)). Combinations of the database search terms were used to search the WHO library.

### 2.4. Study Selection and Inclusion Criteria

The titles, abstracts and if indicated, full text of records from online databases and the WHO library (IRIS) were screened for eligibility. Published studies and reports with original prevalence data on LF infection or morbidity from identified countries were included. Where reports were not available, WHO publications referencing their data were used and recorded.

Only literature available in English and published from 1995 onward was included. There were no restrictions on age, study size, design or power. Any uncertainty regarding the inclusion of a paper was resolved through consensus discussion with other authors. 

### 2.5. Data Collection Process

A standardised data collection form was used to record information from included publications. All data was then entered into a Microsoft Excel spreadsheet for analysis.

### 2.6. Data Items

Extracted data included study characteristics, sampling method and prevalence data. Data were extracted directly from text and tables within the publications. 

Primary outcomes were the prevalence of infection (including measurement method and species) and chronic morbidity (lymphoedema or hydrocoele) in the given population. 

Where interventional studies were included, the prevalence data that were most representative of the population were used. That is, when a whole area participated in MDA external to the study, the post-intervention data were used. When an intervention was implemented only on the study sample, baseline data were used.

### 2.7. Risk of Bias Assessment

A modified bias risk assessment tool was developed to evaluate included studies. It was based on the Crowe and GATE validated critical appraisal tools [20,21]. Risk of bias was assessed by one reviewer on the basis of five independent factors: total sample size (>1000, 300–1000 or <300), sampling of location (representative randomisation, not stated/unclear or non-representative), sampling of participants (representative randomisation, not stated/unclear or non-representative), assessment of infection (internationally accepted methodology, not stated/unclear or non-consensus methods) and assessment of morbidity (independently assessed, not stated/unclear or patient reported). A total risk of bias score was generated by allocating zero, one or two points for each factor. The quality assessment was used to interpret the reliability of each study’s results. Because of the limited number and significant quality variation between studies, the bias assessment was not incorporated directly into the meta-analysis. 

WHO filariasis publications are based on national LF programs annual reports. WHO Regional Program Review Groups (RPRG) assess annual national LF program surveillance reports during their consideration of the country’s request for the donation of MDA drugs. Although general guidelines are provided by WHO, methodology within countries frequently varies substantially and may not be optimal due to poorly resourced programs and frequent personnel changes [22]. Independent assessments of data quality within programs are rarely done. 

### 2.8. Summary Measures and Synthesis of Results

Available data were summarised using percentage prevalence estimates and presented in tables by country and region.

A meta-analysis of primary outcomes from studies was completed using the *metaprop* procedure in STATA version 12.1 to produce overall estimates with exact binomial confidence intervals. A subset meta-analysis of migrant workers in Thailand and India was also done. A random effects model was used to account for the variation in LF prevalence between and within studies.Weighting of studies was done using the method of DerSimonian and Laird [23]. The heterogeneity of data from each meta-analysis was measured using the I^2^ statistic [24].

A map of baseline and most recent LF distribution was generated by combining government prevalence maps found in grey literature. Endemicity was classified at implementation unit level by all countries. Recent maps were available for all countries except Malaysia.

## 3. Results

### 3.1. Study Selection

**Figure 1:**
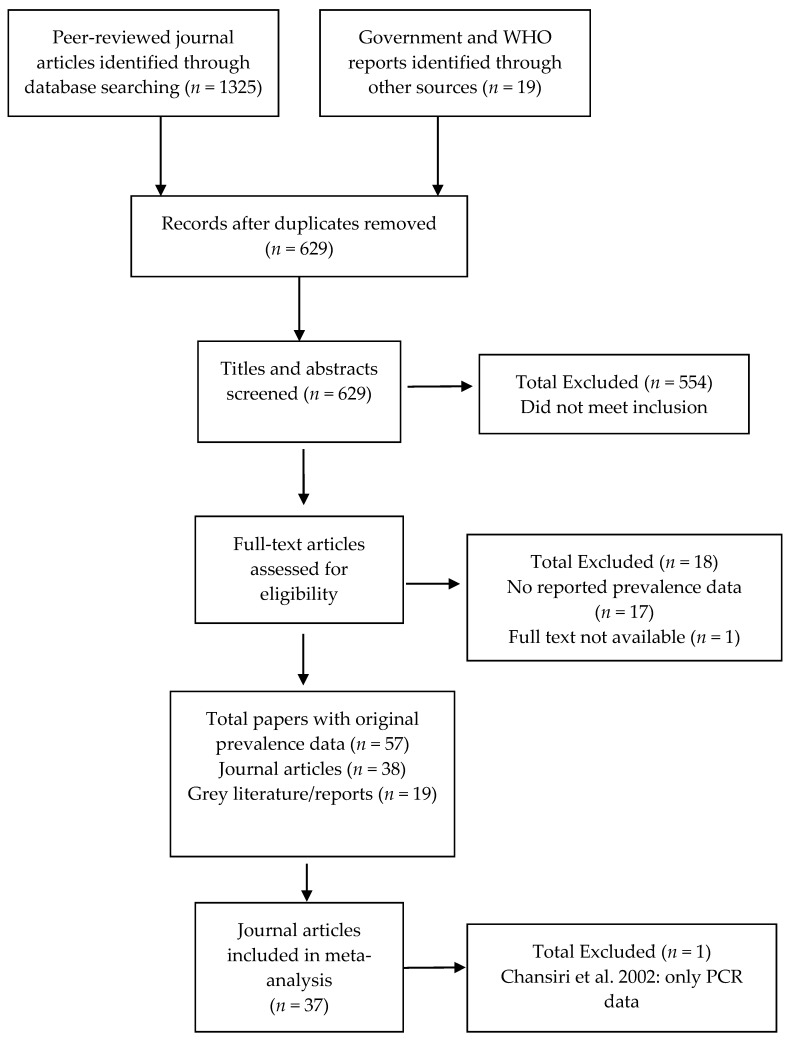
Study selection flow chart.

The search protocol identified 629 papers (Figure 1). Of these, 554 clearly did not meet the inclusion criteria (i.e., were review articles or did not include original prevalence data on infection or morbidity published since 1995 from the identified areas). From the remaining 75 records, 17 were excluded because they did not contain original prevalence data, and one because the full text was not available after contacting the author. The included 57 papers comprised 38 peer-reviewed journal articles and 19 grey literature reports [5,25,26,27,28,29,30,31,32,33,34,35,36,37,38,39,40,41,42,43,44,45,46,47,48,49,50,51,52,53,54,55,56,57,58,59,60,61,62,63,64,65,66,67,68,69,70,71,72,73,74,75,76,77,78,79]. With the exception of Chansiri et al. [30], which contained only polymerase-chain reaction (PCR) infection data, all studies were included in the meta-analysis.

### 3.2. Study Characteristics

The characteristics of the 38 included journal articles are summarised by country in Table 1. This includes 17 from Thailand, eight each from Malaysia and Northeast India, three from Bangladesh and two from Cambodia. There were no studies included from Lao PDR, Myanmar or Vietnam.

All articles described primary studies, and included cross-sectional surveys (CSS) (25 studies, 66%), field diagnostic test evaluation studies (FDE) (8, 21%), field drug trials (FDT) (3, 8%) as well as a longitudinal observational and a retrospective cohort study. Publication year ranged from 1995 to 2016.

Together the included studies assessed 382,274 participants with wide variation in sampling unit and sample size (145 to 232,005). Two studies, Saha et al. (2011) and Krairittichai et al. (2012), contributed substantially to overall participant numbers with sample sizes of 232,005 and 102,090, respectively [44,55]. Eleven of the 17 studies from Thailand assessed Myanmar migrant populations, whilst seven of the eight papers from Northeast India examined those living in tea estates. 

Studies most frequently assessed infection with the traditional thick blood smear (TBS) method (30 studies, 79%), followed by ICT (10, 26%), IgG4 antibody (10, 26%), Og4C3 ELISA (6, 16%) and PCR (3, 8%) tests. One study used urine rather than blood to detect IgG4 antibodies [56]. Almost half of the studies (16, 42%) used multiple methods. Sixteen studies assessed the prevalence of chronic LF morbidity. Of these, seven (44%) assessed both hydrocoele and lymphoedema. 

Table 2 summarises the characteristics of the 19 included grey literature reports. These include 15 WHO publications, three annual national programmatic reports and a report from a non-governmental organisation.With the exception of one WHO report, all papers summarise data from government prevalence surveys conducted as part of their national MDA programs. Whilst only some sample sizes were reported, at least 129,736 individuals were surveyed. Baseline mapping and MDA surveillance surveys predominantly used TBS to diagnose infection, whilst post-MDA TASs used ICT.Some government morbidity information was available for all countries, but the data were very limited.

### 3.3. Risk of Bias within Studies

The risk of bias assessment of included studies is summarised in Table S1. Overall, study quality was suboptimal. Only seven studies (18%) used fully representative sampling methods and consensus data collection methods. 

The sample sizes of published studies were overall acceptable, with 33 studies including greater than 300 participants. However, sample sizes were generally small relative to government surveys. 

The sampling of study location introduced a significant risk of bias. Only ten (26%) studies adequately described the random site selection required for regionally representative data. Of the remaining studies, 21 (55%) did not describe the method/reason for study site selection, and seven (18%) intentionally selected study sites.

Participant sampling within study locations also contributed to risk of bias. Only 18 (47%) studies described random participant selection. Eighteen (47%) studies did not clearly state the method of selection, whilst the remaining two studies excluded those with recent diethylcarbamazine treatment.

With the exception of Chansiri et al., all studies used a consensus method for the detection of LF infection [30]. It is unclear whether the TBS samples in Krairittichai et al. were taken at night, which may have affected the sensitivity of their results [44].

Of the fifteen studies that assessed LF morbidity, only four (25%) described examination methods in detail. Of the remaining papers, eight (50%) provided some detail and four (25%) did not state the method used.

## 4. Prevalence Results and Discussion by Country

### 4.1. Overview

This systematic review and meta-analysis assessed the prevalence estimates for LF infection and morbidity in mainland Southeast Asia, Bangladesh and Northeast India. These estimates are important for the successful implementation and evaluation of elimination programs. Data on the prevalence and distribution of infection are needed to identify and prioritise regions for inclusion in MDA programs. MDA rounds aim to interrupt LF transmission to prevent new infections, eventuating in LF elimination. Prevalence data are then required following MDA rounds to evaluate their effectiveness and reassess the need for further rounds. However, even after transmission has ceased, those with previous infection may, or will have already developed chronic disease manifestations. National programs therefore require data on the morbidity burden in order to implement alleviation programs.

**Figure 2:**
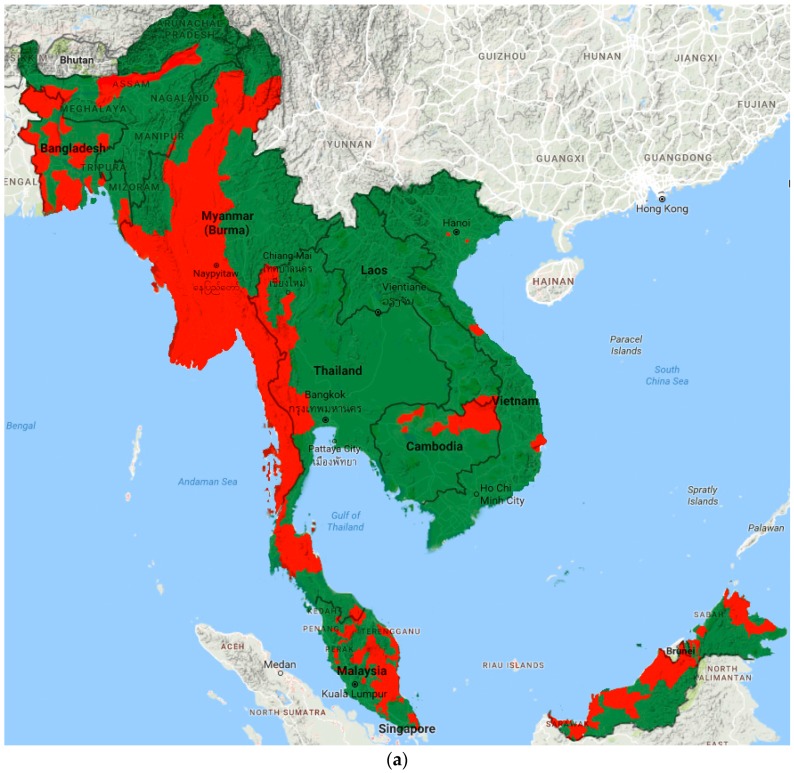

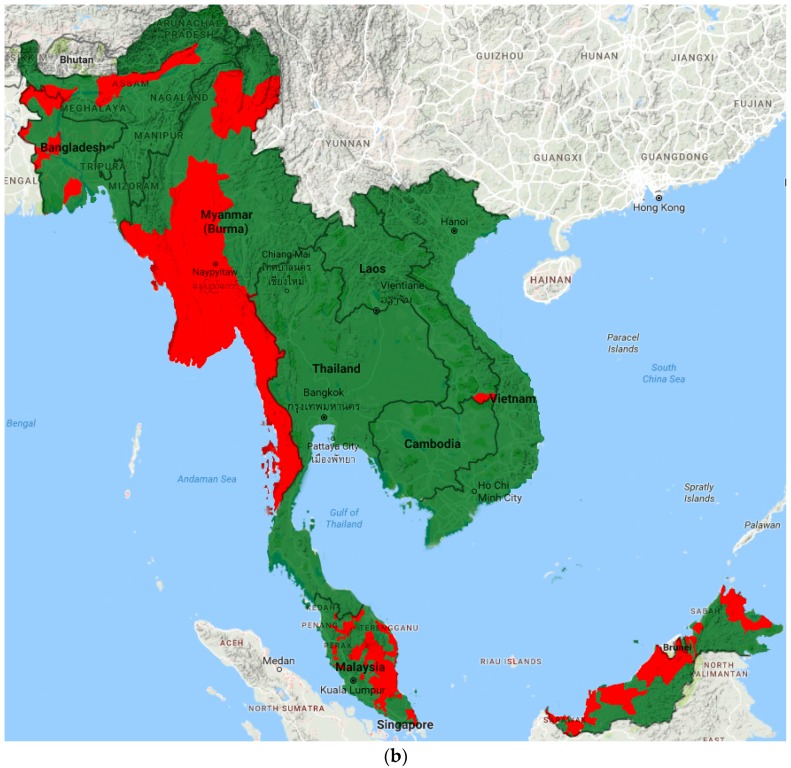
Distribution of LF endemic areas (red): (**a**) at baseline; and (**b**) based on most recent data.

Prevalence data from included studies and grey literature are summarised by country and region in Tables S2–S3. Figure 2 illustrates the most recent distribution of LF compared to that at baseline (pre-MDA).

Exact prevalence estimates cannot be calculated because of the nature of available data. However, we have attempted to estimate the current prevalence of infection and morbidity for each country through assessment of pooled country estimates, grey literature and the quality of available data.

Given the varied sensitivity of detection methods, we defined infection prevalence as low (Mf <0.5%, ICT/Og4C3 <1%, IgG4 <2%), medium (Mf 0.5–1.9%, ICT/Og4C3 1–3.9%, IgG4 2–7.9%), high (Mf 2–3.9%, ICT/Og4C3 4–7.9%, IgG4 8–15.9%) and very high (Mf ≥4%, ICT/IgG4 ≥8%, IgG4 ≥16%). For morbidity, we defined a prevalence of <0.5% as low, Mf 0.5–1.9% as medium, 2–3.9% as high and ≥4% as very high. Baseline prevalence refers to the level of infection prior to the commencement of the national elimination program.

### 4.2. Bangladesh

#### 4.2.1. Results

Grey literature indicates that bancroftian filariasis was endemic in 34 of the 64 districts of Bangladesh during mapping between 2002 and 2004, with an estimated 75.96 million of the country’s 148.77 million at risk [5,65,78]. Fifteen districts were considered low endemic with antigenaemia levels greater than 1% but microfilaraemia less than <0.6% [65,80]. They did not undergo MDA and have now passed two of three TAS, with the last to be completed in 2016 [65]. The remaining 19 districts had initial microfilaraemia prevalences of 0.2–16% [80]. These districts completed MDA rounds in 2014 and have now passed a TAS survey to determine whether MDA can be stopped, with a further TAS planned for 2017 to 2018. National infection prevalence estimates decreased from 1% in 2004 to 0% in 2010 [5]. 

Three studies from 2011–2013 have assessed infection prevalence in the northern endemic states of Nilphamari and Panchargarh [33,55,56]. They have found prevalences by Mf, ICT and IgG4 of 1.13%, 0.31–1.70% and 2.19%, respectively. 

As of 2011, government surveys had identified 23,486 cases of lymphoedema (0.10%) and 65,320 (0.27%) cases of hydrocoele [76]. Two studies have assessed the morbidity prevalence in Bangladesh [33,55]. A large household survey of 232,005 participants reported a lymphoedema prevalence of 0.45%, whilst a smaller survey found a prevalence of lymphoedema and hydrocoele of 2.66% and 4.16% respectively

#### 4.2.2. Discussion

Government data suggests *W. bancrofti* was historically widespread across Bangladesh with a focus in the country’s west. Baseline infection prevalence appeared high to very high, consistent with a previous review and a study of Bangladeshi migrants [15,62]. MDA in Bangladesh appears to have been successful with both government data and recent studies demonstrating low to moderate levels of ongoing infection. The country has now transitioned to post-MDA surveillance and will aim to apply for elimination status after TAS in 2018.

Large-scale household surveys and government data suggest an overall low morbidity burden but a high prevalence of lymphoedema and very high prevalence of hydrocoele in Nilphamari District. As Bangladesh approaches LF elimination, resources should be shifted to further quantifying and tackling the morbidity burden.

### 4.3. Cambodia

#### 4.3.1. Results

Government mapping in 2004 reported 18 endemic districts in four northern and northeastern provinces with 3.61% of the national population at risk (474 800) [5]. Government surveys reported a pre-MDA prevalence of 0.38–2.75% in these provinces (mixed ICT and TBS) [65]. A 2000–2001 study in these endemic provinces showed Mf and ICT prevalence ranging 0–1.13%, and 0–1.94%, respectively [45].

Five rounds of MDA were completed between 2005 and 2009. A post-MDA countrywide serological study in 2012 found a prevalence by IgG4 of 6.60% in this north region and 1.19–1.65% in the rest of the country [52]. All endemic districts passed required TAS by 2015 [65]. In 2016, Cambodia was validated by the WHO as having eliminated LF as a public health problem [2].

In 2001, the government reported 58 cases of LF-related morbidity (40 lymphoedema and 18 hydrocoele) in Cambodia [66]. The highest prevalence was in Stung Treng (10), Takeo (10) and Rattanakiri (9). A study from the same year found the prevalence of lymphoedema and hydrocoele in endemic provinces ranged from 0 to 0.44% and 0 to 2.97% respectively [45].

#### 4.3.2. Discussion

Government baseline mapping indicated *W. bancrofti* and *B. malayi* were endemic at low to moderate levels in four provinces in northern Cambodia. Two representative studies and a prior review confirmed these findings [14]. Cambodia completed post-MDA surveillance in 2015 and WHO validated elimination of LF as a public health problem in 2016.

Whilst LF transmission has now ceased, an overall moderate combined morbidity burden remains in Cambodia. Although the government reported only 58 cases of morbidity in 2001, a representative survey from the same year found a low prevalence of lymphoedema (0.34%) and moderate prevalence of hydrocoele (1.64%) across the four endemic provinces. When the study’s results are extrapolated using 1998 census population data [81], case estimates for lymphoedema and hydrocoele are 2800–17,382 and 8474–27,461 in the four provinces, respectively. Now that MDA is complete, efforts in Cambodia need to shift to assessing and alleviating the morbidity burden in the country.

### 4.4. Northeast India

#### 4.4.1. Results

Filariasis is endemic in 250 districts across 20 states in India, with 617 million individuals at risk [78]. Assam is considered the only endemic state in Northeast India [5]. 

Baseline government mapping from 2004 illustrates a line of endemic districts from the Bangladesh to the Myanmar border with a prevalence of 0–5% [5].

Studies prior to the commencement of MDA in 2004 found a bancroftian Mf prevalence of 0.61–10.27% and 0.45–1.79% in tea-estate and non-tea-estate populations, respectively [32,37,38,39,40,51]. Sub-group meta-analysis showed a significantly higher Mf prevalence in populations living in tea-estates (6.11%, 95%CI 3.49–9.41%) compared to those who did not (0.88%, 0.3–1.54) (*p* = 0.000). A more recent study found persistent microfilaraemia of 7.41% in a tea-estate following six rounds of MDA [41]. The government reports that the national infection prevalence has decreased from 1.24% in 2004 to 0.35% in 2011 [5].

The prevalence of lymphoedema and hydrocoele found in tea-estate populations was 0.12–0.72% and 1.01–8.96%, respectively [32,37,39,40,41,51]. One study assessed morbidity in a non-tea-estate community and found no clinical cases [40].

#### 4.4.2. Discussion

Available data indicate that Assam is the only LF-endemic state in Northeast India. Baseline mapping and studies demonstrated very high levels of *W. bancrofti* infection amongst those living in tea-estates and a moderate prevalence in non-tea estate populations. Tea-estates are predominantly composed of workers who migrated from states such as Bengal, Bihar, Odisha, Uttar Pradesh, Madhya Pradesh, Tamil Nadu and Jharkhand as part of the British tea trade in the 19th and early 20th century. Whilst national infection prevalence has progressively declined since the commencement of the MDA program in 2004, a recent study found very high levels of persisting microfilaraemia in a tea-estate following six rounds of MDA [41]. Reports of missed MDA rounds in seven districts in 2009, as well as suboptimal drug coverage may account for the ongoing LF transmission in the region [63,74,75]. Further rounds of MDA with sufficient coverage and uptake are required.

Multiple representative studies have demonstrated an overall moderate burden of disease in the tea-estate population of Assam with a low prevalence of lymphoedema but very high prevalence of hydrocoele. Insufficient datawere available to assess the morbidity burden in the non-tea estate population. In addition to ongoing MDA, programmatic efforts should focus on assessing the morbidity burden in non-tea populations and implementing alleviation programs.

### 4.5. Lao PDR

#### 4.5.1. Results

No studies on the prevalence of LF in Lao PDR were found.Grey literature indicates that *W. bancrofti* is only endemic in the southern Attapeu and Sekong Provinces of Lao PDR with 137,000 individuals at risk [65,66,72,79]. Mapping of the Attapeu’s five districts in 2009 showed an antigenaemia prevalence ranging 1.9–27.4% [65]. MDA commenced in Attapeu in one district in 2008 and was expanded to all five in 2009. Only one village in Sekong Province is endemic with a prevalence of 6% [65]. Two rounds of focused MDA have been completed in this and surrounding villages.

Only one case of lymphoedema and no cases of hydrocoele have been reported in the country [66].

#### 4.5.2. Discussion

Government data from Lao PDR suggest that *W. bancrofti* is focussed in Attapeu province (along the Cambodian border) and parts of the adjacent Sekong province. Infection prevalence was initially considered low to moderate as noted by previous reviews [1,11,14,15]. However, further baseline surveys in 2009 found very high levels of antigenaemia in Attapeu province. Since the commencement of widespread MDA, infection prevalence appears to have decreased to low to moderate levels, however no independent studies are available to validate government data.

Insufficient dataare available to assess the morbidity burden in Lao PDR. Given the infection prevalence in Attapeu and morbidity burden in neighbouring Cambodia, numerous cases would be expected. Further studies are urgently needed to validate government data and assess the morbidity burden in Lao PDR.

### 4.6. Malaysia

#### 4.6.1. Results

WHO reports indicate that 116 of the 994 implementation units (subdistricts and districts) are endemic across Peninsular Malaysia, Sabah and Sarawak with 1.12 million people at risk [72,79]. Subperiodic *B. malayi* accounts for the majority of cases, with 2% caused by *W. bancrofti*. Government data suggest Mf prevalence had decreased from 1% to 0.2% in Peninsular Malaysia and 5.5% to 1.5% in Sabah and Sarawak from the 1980s until the commencement of the National MDA Program in 2004 [66,67]. 

Pre-MDA studies of the local population in Peninsular Malaysia, Sabah and Sarawak found a prevalence of *B. malayi* Mf of 0.26–23.85%, 20.69% and 0.90%, respectively [31,34,35,46,53].

Since 2004, Malaysia has completed five rounds of MDA in Peninsular Malaysia and seven rounds in Sabah and Sarawak. As of 2014, only five IUs were still conducting MDA rounds with the remainder commencing TAS [79]. More recent studies in Peninsular Malaysia have found a prevalence of Mf and IgG4 of 0 and 2.16% respectively [25,54].

No data on the prevalence of LF-associated morbidity in the local Malaysian population were found.

#### 4.6.2. Discussion

*Brugia malayi* (and to a lesser extent *W. bancrofti*) was widely endemic across Peninsular Malaysia, Sabah and Sarawak. Government surveys reported a low to moderate baseline infection prevalence. Whilst pre-MDA studies showed greater levels of infection than government data, most were not representative. These studies likely reflected the presence of highly-endemic foci in parts of Peninsular Malaysia and Sabah, rather than a high baseline prevalence. More recent surveys suggest infection levels are declining in Malaysia, although one study was conducted in a known low-endemic area [54]. Whilst recent government data are lacking, a 2016 xenomonitoring survey across five states in Peninsular Malaysia and Sabah found no active transmission [82], suggesting a likely low level of infection and supporting Malaysia’s progress toward LF elimination. Further studies are required to assess current infection prevalence and the need for ongoing MDA.

Although there are no data on the morbidity prevalence in the local Malay population, a significant burden of lymphoedema is expected given the previously highly endemic foci of *B. malayi* infection. Studies assessing the morbidity burden are needed to fill this knowledge gap.

### 4.7. Myanmar

#### 4.7.1. Results

Bancroftian filariasis is endemic in 45 of the 65 districts of Myanmar with 85.5% of the population at risk [5]. No published prevalence studies on Myanmar were found. One study described in a WHO report assessed filarial antigenaemia prevalence in 1997 [73]. One hundred individuals were tested by ICT from seventy randomly selected townships across 14 districts. The central and western dry zone was highly endemic (20–30%) with the northern, eastern and southern areas less endemic or free from filariasis. Most recent government microfilaraemia prevalence data for the western Sagaing, Magway, Rakhine and Chin regions range from 0–65% [68,69,70]. Prevalence in Mandalay, Kayin and Yangon regions ranges from 0–2% [68,69,70]. Mean national prevalence has reduced from 7.1% in 2001 to 2.7% in 2011 [5]. Myanmar commenced its MDA Program in 2001. As of 2014, the program had been scaled-up to 22 of the 24 endemic districts.

One government survey assessed self-reported morbidity in 280 000 individuals in Sagaing region in 2004 and identified 520 cases of lymphoedema (0.19%) and 827 cases of hydrocoele (0.59%) [68].

#### 4.7.2. Discussion

Baseline surveys suggest that *W. bancrofti* were widely endemic in the low-lying parts of Myanmar with over 85% of the population at risk. They suggested very high baseline levels of infection in the central and western dry zones, consistent with that seen in Myanmar migrants in Thailand. Meanwhile, the northern, eastern and southern areas were less endemic or free from filariasis. Government data suggest declining levels of infection in Mandalay, Kayin and Yangon states since the commencement of MDA, but very high rates of persisting infection in the western states of Sagaing, Magway, Rakhine and Chin. As Myanmar continues to expand its MDA program, independent studies are required to validate government prevalence data.

The only morbidity survey found low levels of self-reported lymphoedema and moderate levels of hydrocoele in Sagaing region [68]. However, morbidity self-reporting questionnaires are not a sensitive or specific indicator of clinical disease [45,83]. The significant morbidity found in Myanmar migrants in Thailand suggests that the true morbidity prevalence is likely much higher. Representative prevalence studies are required to elucidate the morbidity burden in Myanmar.

### 4.8. Thailand

#### 4.8.1. Results

Baseline government mapping in 2001 found filariasis to be endemic in two foci in Thailand with 160,000 individuals at risk [5]. *Wuchereria bancrofti* was present in five provinces along the Thai-Myanmar border, whilst *B. malayi* was endemic along the southern Thai peninsula. Studies in provinces along the Myanmar border between 1998 and 2003 found an infection prevalence of 1.01–10.20%, 13.3–26.42%, 21.89–36.79% and 53.93% by TBS, ICT, Og4C3 and IgG4, respectively [26,27,29,49,50]. Meanwhile, studies conducted along the Thai peninsula between 2001 and 2006 reported a prevalence of *B. malayi* infection of 0–2.00% and 8.00–23.67% by TBS and IgG4 [36,43,59].

The 11 studies which assessed infection in Myanmar migrants living in Thailand found a prevalence of 0–5.83%, 0.2–13.57%, 4.0–23.98%, and 2.73–42.32% by TBS, ICT, Og4C3 and IgG4, respectively [26,28,29,42,43,48,57,58,60,61]. 

Thailand commenced an elimination program in 2002. Mass drug administration was completed in 2007 in all provinces except Narathiwatt, which extended rounds until 2011. Thailand commenced the process of verifying LF elimination in 2012 after all areas passed TAS. National mean data suggest infection prevalence has decreased from 0.77% in 2003 to 0.09% in 2010 [5]. No post-MDA prevalence studies on the local population were identified. 

Available studies have found the prevalence of lymphoedema and hydrocoele in the local population in endemic areas was 1.2% and 0–8.15% [27,29,36]. Studies of Myanmar migrants found a prevalence of hydrocoele of 8.62–16.13% [29,48]. As of 2011, the government had 200 identified cases of lymphoedema [77]. 

#### 4.8.2. Discussion

Two highly endemic LF foci previously existed in Thailand. *W. bancrofti* was present along the Thai-Myanmar border, whilst *B. malayi* was endemic along the southern Thai peninsula. Government surveys suggest that LF transmission has ceased following MDA, but no studies have independently verified this. 

The influx of LF-infected Myanmar migrants into Thailand, combined with a documented ability of Thai *Culex quinquefasciatus* mosquitoes to transmit the Myanmar strain of *W. bancrofti* has raised concerns regarding the potential for re-introduction of transmission [84,85]. The Thai government has acknowledged this and expanded its MDA program to include registered migrant workers from Myanmar and Laos [57,86]. However, the considerable number of unregistered migrants still poses a threat to elimination efforts. 

Whilst the government had only identified 200 cases of lymphoedema in 2011, available studies suggest a potentially moderate prevalence of lymphoedema and very high prevalence of hydrocoele in previously endemic areas. However the representativeness of these surveys is uncertain, indicating the need for further studies to establish the true disease burden in these areas.

### 4.9. Vietnam

#### 4.9.1. Results

No peer-reviewed studies from Vietnam were identified. Government data suggest filariasis was endemic in six of Vietnam’s eight regions during 1960–2000 [66]. However, by the start of the National Elimination Program in 2003, only the Red River delta, north-central coast and central-southern coast regions remained endemic despite no MDA [65,66]. Within these regions, 12 districts were considered endemic and six selected for MDA with a total population at-risk of 675,000. *Brugia malayi* predominates in the north with *W. bancrofti* in the south [65,66]. 

Baseline prevalence in endemic districts in 2002 ranged from 0 to 3.6% [65,66]. Vietnam completed its MDA program in 2011 and passed TAS in all districts in 2015. It has subsequently applied to the WHO for LF elimination confirmation [65].

A government clinical survey in 2002 in 77 districts reported 570 cases of limb morbidity and 47 cases of hydrocoele [66]. A later survey in 2012 identified 489 morbidity cases in five provinces [65].

#### 4.9.2. Discussion

Filariasis was historically widespread in Vietnam with *Brugia malayi* predominating in the north and *W. bancrofti* in the south. However by the commencement of the MDA Program, it had become confined to the Red River delta, north-central coast and central-southern coast regions. These findings are consistent with those found in prior reviews [14,15,16]. It has been suggested that the reduction in infection prevalence prior to MDA was likely the result of improvements in housing and living conditions, man-made ecological changes, the use of bed-nets and individual case treatment [16,65]. These changes appear to have resulted in low baseline infection prevalence levels by the start of the MDA with the exception of Khanh Vinh and Ninh Hoa districts, where prevalence remained high. Government data suggest that since MDA, LF transmission has now ceased, although no independent studies have yet validated this.

Whilst government surveys suggest a relatively few cases of morbidity, the number of participants surveyed is unknown, and therefore prevalence estimates cannot be made. Given filariasis was historically widespread with some highly endemic foci, the true morbidity burden may be higher [16]. As Vietnam approaches LF elimination, studies are needed to validate government progress and assess the morbidity burden in the country.

### 4.10. Overall Prevalence Estimates for Mainland Southeast Asia

#### 4.10.1. Meta-Analysis Results

A random effects meta-analysis of available infection and morbidity data was completed. Figure 3, Figure 4, Figure 5, Figure 6 and Figure 7 and Figures S1–S9 show the point estimates with 95% exact binomial confidence intervals (95% CI) for each meta-analysis ordered by country and study date. Substantial heterogeneity between surveys was demonstrated by I^2^ values, between 83.57% and 98.74%. 

For microfilaraemia, 30 studies testing 133,747 individuals estimated a pooled prevalence of *W. bancrofti* Mf of 1.77% (1.00–2.74%), *B. malayi* Mf of 0.36% (0.07–0.82%) and combined Mf of 2.64% (1.71–3.74%). For ICT antigenaemia, 10 studies testing 7237 individuals estimated a pooled prevalence of 4.48% (1.97–7.87%). For IgG4 antibodies, 10 studies testing 14,339 individuals estimated a pooled prevalence of 7.08% (3.63–11.54%). 

For combined morbidity, 16 studies testing 256,591 individuals estimated a pooled prevalence of 1.34% (0.81–1.97%). For hydrocoele, nine studies testing 4179 estimated a pooled prevalence of 3.84% (2.11–6.03%). For lymphoedema, nine studies testing 240,987 individuals estimated a pooled prevalence of 0.49% (0.24–0.80%). 

#### 4.10.2. Discussion

Meta-analysis showed an overall high prevalence of infection (Mf: 2.64%, antigenaemia: 4.48%) and moderate burden of morbidity (1.34%) in the region. However, pooled estimates and sub-group analyses by country are biased by the significant differences in location, time, design, diagnosis method, sampling unit and representativeness of the included studies, and should therefore be interpreted with caution. Despite this, meta-analyses show a general decline in infection prevalence with time and a higher prevalence of hydrocoele compared to lymphoedema across all countries (overall hydrocoele:lymphoedema ratio of 7.84:1). 

In the context of pooled country estimates, grey literature and the quality of available data, we conclude that Lao PDR, Myanmar and Northeast India demonstrate ongoing evidence of LF transmission that will require multiple further rounds of MDA. Bangladesh, Malaysia, Thailand and Vietnam appear close to having eliminated LF, whilst Cambodia has already achieved elimination status. We estimated that the burden of morbidity is likely high in Thailand, moderate in Cambodia, Myanmar, and Northeast India, and low in Bangladesh. There was insufficient evidence to accurately estimate the disease burden in Lao PDR, Malaysia or Vietnam.

These results indicate that whilst considerable progress toward LF elimination has been made, there remains a significant filariasis burden in the region. The results will assist policy makers to advocate and budget for future MDA and morbidity control programs. 

### 4.11. Limitations

This systematic review was hindered by limitations at the study and review level. The overall quality of primary studies was suboptimal. Only 18% of studies used fully representative sampling methods and consensus data collection methods. Some of the studies were out-dated because one or more MDA rounds had occurred since data collection. The quality of grey literature further hindered data analysis. Available reports frequently omitted sample sizes or gave prevalence data in percentage ranges, making further analysis difficult.The uncertain methodology of government surveys further complicated grey literature assessment. Whilst national programs follow the WHO guidelines, there is often considerable variation in sampling and data collection practices in the field [22]. No published studies were available from Lao PDR, Myanmar or Vietnam to compare with government data. 

Incomplete literature retrieval led to limitations at the review level. Original copies of government annual reports were obtained only from Myanmar, although use of WHO reports alleviated this deficiency to some extent. Retrieval of these data would provide a more complete picture of filariasis prevalence in these countries.

The lack of representative and recent studies in many of the countries placed reliance on government sources for current prevalence data. Whilst sufficient information on infection was available to produce country estimates, data on the morbidity burden are notably lacking. This indicates the substantial need for further studies, with a particular focus on morbidity, to more accurately assess the current LF prevalence in the region. 

It is important to note that the findings and conclusions in this paper are based solely on published prevalence data. It is therefore possible that they may not truly reflect the actual situation in these countries. 

## 5. Conclusions

Considerable progress has been made toward the LF elimination in mainland Southeast Asia, Bangladesh and Northeast India. Five of the eight countries reviewed are close to eliminating, or have eliminated, LF infection. The remaining three countries will require increasing support if they are to achieve LF elimination by 2020. The significant morbidity burden in the region requires increasing and urgent attention. Further studies are needed to more accurately assess the morbidity prevalence and implement desperately needed alleviation programs.

## Figures and Tables

**Figure 3:**
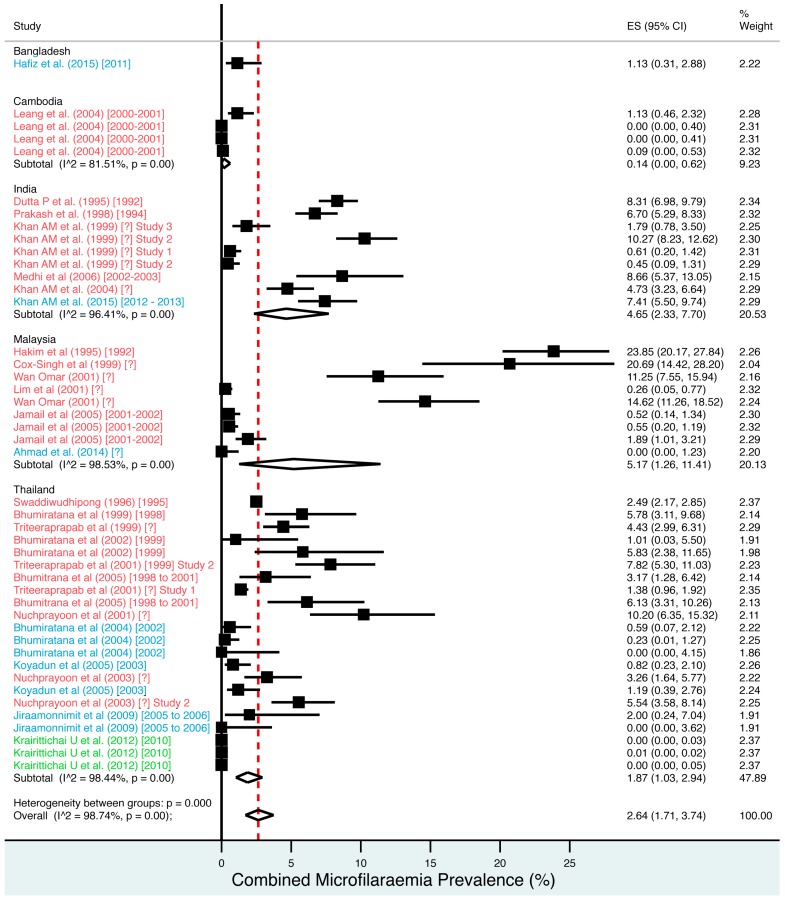
Percentage estimates of combined microfilaraemia prevalence by country and year. ES: prevalence estimate. Red-dashed line: overall estimate. Diamond: subgroup estimate. Horizontal line: 95% CI. Red study: pre-MDA, blue study: mid-MDA, green study: post-MDA.

**Figure 4:**
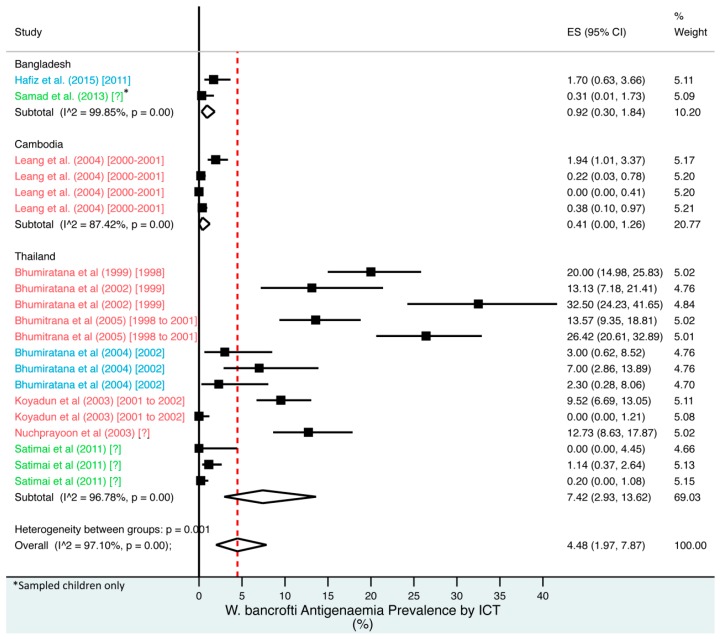
Percentage estimates of ICT antigenaemia prevalence by country and year. ES: prevalence estimate. Red-dotted line: overall estimate. Blue diamond: sub-group estimate. Horizontal line: 95% CI. Red study: pre-MDA, blue study: mid-MDA, green study: post-MDA.

**Figure 5:**
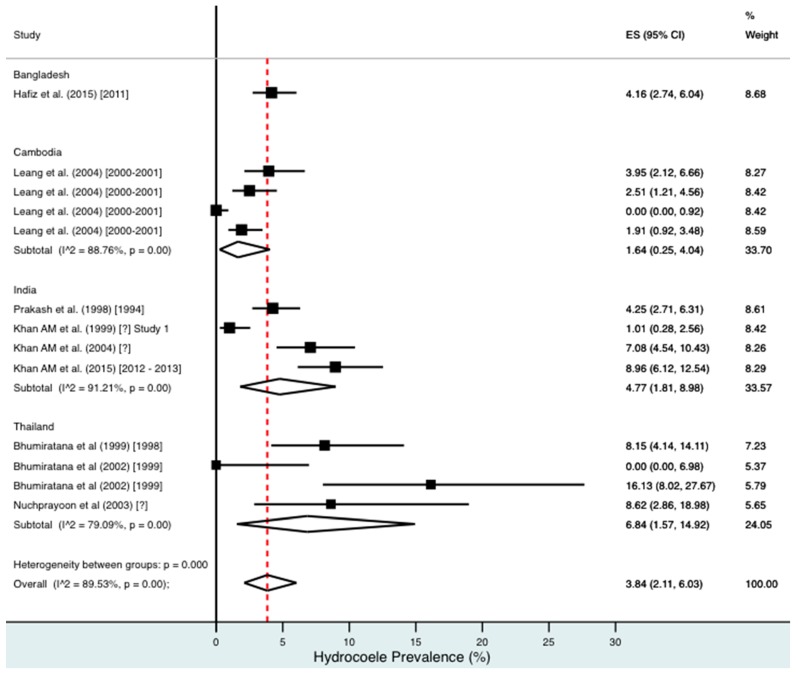
Percentage estimates of hydrocoele prevalence by country and year. ES: prevalence estimate. Red-dotted line: overall estimate. Diamond: sub-group estimate. Horizontal line: 95% CI.

**Figure 6:**
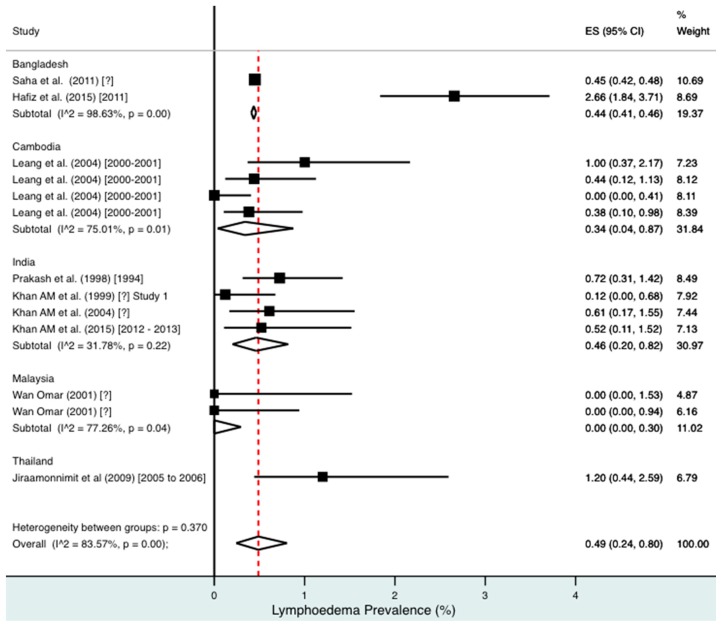
Percentage estimates of lymphoedema prevalence by country and year. ES: prevalence estimate. Red-dotted line: overall estimate. Diamond: sub-group estimate. Horizontal line: 95% CI.

**Figure 7:**
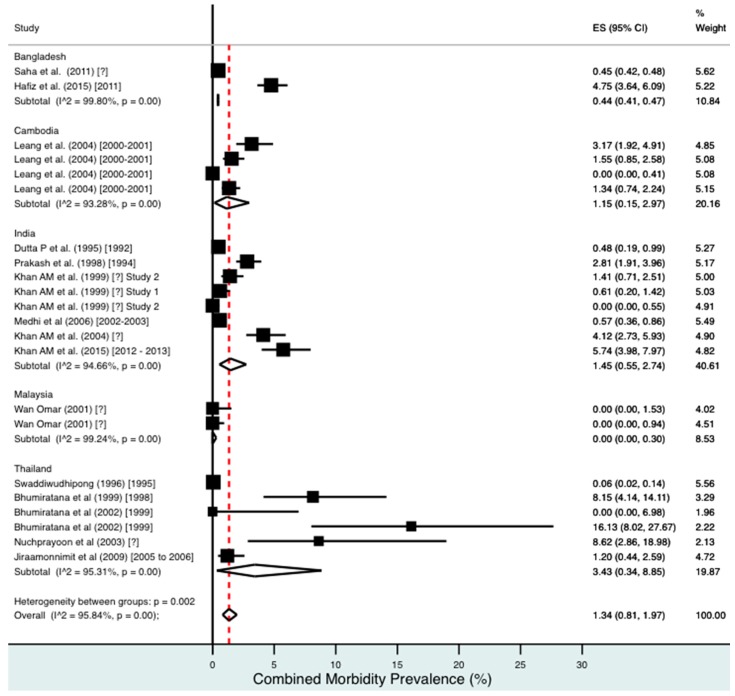
Percentage estimates of combined morbidity prevalence by country and year. ES: prevalence estimate. Red-dotted line: overall estimate. Diamond: sub-group estimate. Horizontal line: 95% CI.

**Table 1:** Included peer-reviewed journal articles.

Study (Publication Date) [Study Period] [Ref.]	Study Design	Sampling Population/Unit (Age of Participants)	Sample Size	Diagnostic Method	
				Infection	Morbidity
*Bangladesh*					
Hafiz et al. (2015) (2011) [33]	CSS ^b^	Households in 30 villages (≥10)	1242	Mf, ICT	Hyd./Lymph. ^h^
Saha et al. (2011) [NS ^g^] [55]	CSS	Households in 19 unions (≥1)	232,005	-	Lymph.
Samad et al. (2013) [NS] [56]	FDE ^a^	School children (5–10)	319	ICT, IgG4 ^e^	-
*Cambodia*					
Leang et al. (2004) (2000–2001) [45]	CSS	83 villages (≥1)	3468	Mf, ICT	Hyd./Lymph.
Priest et al. (2016) (2012) [52]	CSS	2200 households (women 15–39)	2150	IgG4	-
*Northeast India*					
Dutta et al. (1995) (1992) [32]	CSS	Individuals in 1 tea estate (≥1)	1553	Mf	NS
Khan et al. (1999) [NS] Study 1 [37]	CSS	Individuals in 1 tea estate (≥1)	821	Mf	Hyd./Lymph.
Khan et al. (1999) [NS] Study 2 [40]	CSS	2 communities: tea workers and non-tea workers (≥1)	1446	Mf	NS
Khan et al. (1999) [NS] Study 3 [38]	CSS	1 weaving community (≥1)	446	Mf	-
Khan et al. (2004) [NS] [39]	CSS	Individuals in 1 tea estate (≥1)	656	Mf	Hyd./Lymph.
Khan et al. (2015) (2012–2013) [41]	CSS	Individuals in 1 tea estate (infection ≥2, morbidity ≥18)	634	Mf	Hyd./Lymph.
Medhi et al. (2006) (2002–2003) [47]	CSS	Households in 8 tea estates (≥1)	4016	Mf	Hyd./Lymph.
Prakash et al. (1998) (1994) [51]	CSS	Households in 1 tea estate (≥1)	1105	Mf	Hyd./Lymph.
*Malaysia*					
Ahmad et al. (2014) [NS] [25]	CSS	Households/schools on 1 island (≥1)	298	Mf	-
Cox-Singh et al. (1999) [NS] [31]	CSS	2 districts (NS)	145	Mf, PCR	-
Hakim et al. (1995) (1992) [34]	FDT ^c^	2 villages (≥6 months old)	499	Mf	-
Jamail et al. (2005) (2001–2002) [35]	FDE	7 districts(≥1)	2545	Mf, IgG4	-
Lim et al (2001) [NS] [46]	FDE	5 villages and 2 schools (≥1)	1134	Mf, IgG4	-
Rahmah et al. (2003) [NS] [53]	FDE	16 schools (7–12)	5138	IgG4	-
Rahmah et al. (2010) [NS] [54]	FDE	School children (6–10)	973	IgG4	-
Wan Omar et al. (2001) [NS] [62]	FDE	Migrant workers in palm oil estates (WA ^i^)	630	Mf	Lymph.
*Thailand*					
Bhumiratana et al. (2004) (2002) [28]	FDT	Myanmar workers (WA ^i^)	860	Mf, ICT, Og4C3	-
Bhumiratana et al. (2005) (1998–2001) [26]	CSS	Myanmars and Thais in multiple villages (≥1)	433	Mf, ICT, Og4C3	-
Bhumiratana et al. (1999) (1998) [27]	FDE	Multiple villages (≥1)	225	Mf, ICT	Hyd.
Bhumiratana et al (2002) (1999) [29]	CSS	1 village (≥1)	219	Mf, ICT	Hyd.
Chansiri et al. (2002) (1997–2001) [30]	CSS	Migrant workers in 4 provinces (WA ^i^)	1299	PCR	-
Jiraamonnimit et al. (2009) (2005 to 2006] [36]	LO ^d^	3 provinces (≥7)	500	Mf, IgG4	Lymph.
Koyadun et al. (2003) (2001–2002) [43]	FDT	Myanmar migrants and Thais in 3 districts (≥15)	660	ICT	-
Koyadun et al. (2005) (2003) [42]	CSS	Myanmar workers (≥10)	904	Mf	-
Krairittichai et al. (2012) (2010) [44]	RCS ^f^	Migrant workers at 1 hospital (WA ^i^)	102,090	Mf	-
Nuchprayoon et al. (2003) [NS] Study 1 [48]	FDE	Myanmar workers at 2 factories (WA ^i^)	337	Mf, ICT, Og4C3	Hyd.
Nuchprayoon et al. (2003) [NS] Study 2 [49]	CSS	2 villages (≥1)	433	Mf, Og4C3, IgG4	-
Nuchprayoon et al. (2001) [NS] [50]	CSS	1 sub-district (≥1)	196	Mf, Og4C3, PCR	-
Satimai et al. (2011) [NS] [57]	CSS	Myanmar migrants and Thais in 2 provinces (≥1)	1031	ICT, IgG4	-
Swaddiwudhipong et al. (1996) (1995) [58]	CSS	Myanmar workers and their families (≥1)	8377	Mf	NS
Triteeraprapab et al. (1999) [NS] [61]	CSS	Myanmar workers in 6 industrial plants (WA ^i^)	654	Mf	-
Triteeraprapab et al. (2001) [NS] Study 1 [59]	CSS	4 districts in 1 province (≥1)	2462	Mf	-
Triteeraprapab et al. (2001) (1999) Study 2 [60]	CSS	Myanmar migrants in 1 community (≥2)	371	Mf, Og4C3, IgG4	-

^a^ Field diagnostic test evaluation. ^b^ Cross-sectional survey. ^c^ Field Drug trial. ^d^ Longitudinal observational study. ^e^ Urine IgG4. ^f^ Retrospective cohort study. ^g^ Not stated. ^h^ Hydrocoele/lymphoedema. ^i^ Working age.

**Table 2:** Included grey literature.

Reports [Ref.]	Year	Sample Size	Diagnostic Method	
			Infection	Morbidity
*Annual Reports*				
Myanmar Ministry of Health. National Program to Eliminate LF: Annual Reports [68,69,70]	2004	23,668	Mf	-
	2011	10,845	Mf	-
	2012	14,649	Mf	Hyd./Lymph. ^c^
*World Health Organization Reports*				
WHO SEARO: Elimination of lymphatic filariasis in the Southeast Asia Region. Reports of the 1st, 7th, 8th, 9th, 10th Meeting of the Regional Program Review Group. [74,75,76,77,78]	2005	NS ^b^	Mf (ICT) ^a^	Hyd./Lymph.
	2010			
	2011			
	2012			
	2013			
WHO Regional Office for Southeast Asia: Elimination of lymphatic filariasis in the Southeast Asia Region. Reports of the 5th and 8th Meeting of the Regional Program Managers. [63,64]	2006	NS	Mf (ICT) ^a^	Hyd./Lymph.
	2011			
WHO Regional Office for Southeast Asia: Towards eliminating lymphatic filariasis: Progress in the Southeast Asia Region (2001–2011). [5]	2013	NS	Mf	Hyd./Lymph.
WHO Regional Office for Southeast Asia: Regional Strategic Plan for Elimination of Lymphatic Filariasis (2004–2007). [71]	2004	NS	Mf	Hyd./Lymph.
WHO Western Pacific Region: First Mekong-Plus Program Managers Workshop on Lymphatic Filariasis and Other Helminthiasis. [66]	2009	Cambodia: 23,705 Lao PDR: 9286 Vietnam: 18,302	Mf/ICT	Hyd./Lymph.
WHO Regional Office for the Western Pacific. Reports of the 13th and 14th Meeting of the Western Pacific Regional Program Review Group on Neglected Tropical Diseases. [72,79]	2013	NS	Mf/ICT	Hyd./Lymph.
	2014			
WHO Malaysia Office. Country Cooperation Strategy 2009–2013. [67]	2010	NS	Mf	-
WHO: Meeting of the Neglected Tropical Disease Strategic and Technical Advisory Group’s Monitoring and Evaluation Subgroup on Disease Specific Indicators. [80]	2014	NS	Mf	-
UNDP/World Bank/WHO/UNICEF. Research on rapid geographical assessment of Bancroftian filariasis. [73]	1997	7000	ICT	-
*Non-Governmental Organization Reports*				
Family Health International 360 and USAID: End Neglected Tropical Diseases in Asia Final Report. [65]	2015	Cambodia: 18,809 Lao PDR:3472 Vietnam: NS	Mf/ICT	Hyd./Lymph.

^a^ ICT only used in one survey. ^b^ Not stated. ^c^ Hydrocoele/lymphoedema.

## Supplementary Materials

The following are available online at www.mdpi.com/2414-6366/2/3/32/s1. Table S1: Quality Assessment of Included Peer-Reviewed Journal Articles; Table S2: Infection and Morbidity Prevalence by Country and Region in Peer-Reviewed Journals; Table S3: Infection and Morbidity Prevalence by Country and Region in Grey Literature; Figure S1: Percentage estimates of *W. bancrofti* microfilaraemia prevalence by country and year; Figure S2: Percentage estimates of *B. malayi* microfilaraemia prevalence by country and year; Figure S3: Percentage estimates of Og4C3 antigenaemia prevalence by country and year; Figure S4: Percentage estimates of IgG4 antibody prevalence by country and year; Figure S5: Percentage estimates of combined microfilaraemia prevalence in Northeast India; Figure S6: Percentage estimates of combined microfilaraemia prevalence in Thailand; Figure S7: Percentage estimates of ICT antigenaemia prevalence in Thailand; Figure S8: Percentage estimates of hydrocoele prevalence in Thailand; Figure S9: Percentage estimates of combined morbidity prevalence in Thailand. Figure S10: PRISMA checklist.

## References

[1] World Health Organization (2010). WHO Global Programme to Eliminate Lymphatic Filariasis: Progress Report for 2000–2009 and Strategic Plan 2010–2020.

[2] World Health Organization (2016). Global programme to eliminate lymphatic filariasis: Progress report, 2015. Wkly. Epidemiol. Record.

[3] Ramaiah K., Ottesen E.A. (2014). Progress and impact of 13 years of the global programme to eliminate lymphatic filariasis on reducing the burden of filarial disease. PLoS Negl. Trop. Dis..

[4] Murray C.J., Barber R.M., Foreman K.J., Ozgoren A.A., Abd-Allah F., Abera S.F., Aboyans V., Abraham J.P., Abubakar I., Abu-Raddad L.J. (2015). Global, regional, and national disability-adjusted life years (DALYs) for 306 diseases and injuries and healthy life expectancy (HALE) for 188 countries, 1990–2013: Quantifying the epidemiological transition. Lancet.

[5] World Health Organization: Regional Office for South-East Asia (2013). Towards Eliminating Lymphatic Filariasis: Progress in the South-East.Region. (2001–2011).

[6] Weil G.J., Jain D.C., Santhanam S., Malhotra A., Kumar H., Sethumadhavan K.V.P., Liftis F., Ghosh T.K. (1987). A monoclonal antibody-based enzyme immunoassay for detecting parasite antigenemia in bancroftian filariasis. J. Infect. Dis..

[7] Weil G.J., Lammie P.J., Weiss N. (1997). The ICT filariasis test: A rapid-format antigen test for diagnosis of bancroftian filariasis. Parasitol. Today.

[8] Gass K., de Rochars M.V.B., Boakye D., Bradley M., Fischer P.U., Gyapong J., Itoh M., Ituaso-Conway N., Joseph H., Kyelem D. (2012). A multicenter evaluation of diagnostic tools to define endpoints for programs to eliminate bancroftian filariasis. PLoS Negl. Trop. Dis..

[9] Lammie P.J., Weil G., Noordin R., Kaliraj P., Steel C., Goodman D., Lakshmikanthan V.B., Ottesen E. (2004). Recombinant antigen-based antibody assays for the diagnosis and surveillance of lymphatic filariasis—A multicenter trial. Filaria J..

[10] Rebollo M.P., Bockarie M.J. (2014). Shrinking the lymphatic filariasis map: Update on diagnostic tools for mapping and transmission monitoring. Parasitology.

[11] World Health Organization (2013). WHO Global Programme to Eliminate Lymphatic Filariasis: Practical Entomology.

[12] World Health Organization (2017). Validation of Elimination of Lymphatic Filariasis as A Public Health Problem.

[13] World Health Organization (2012). Transmission Assessment Surveys in the Global Programme to Eliminate Lymphatic Filariasis: WHO Position Statement.

[14] Sudomo M., Chayabejara S., Duong S., Hernandez L., Wu W.P., Bergquist R. (2010). Elimination of lymphatic filariasis in Southeast Asia. Adv. Parasitol..

[15] Michael E., Bundy D.A.P., Grenfell B.T. (1996). Re-assessing the global prevalence and distribution of lymphatic filariasis. Parasitology.

[16] Meyrowitsch D.W., Nguyen D.T., Hoang T.H., Nguyen T.D., Michael E. (1998). A review of the present status of lymphatic filariasis in Vietnam. Acta Trop..

[17] Moher D., Liberati A., Tetzlaff J., Altman D.G. (2009). Preferred reporting items for systematic reviews and meta-analyses: The PRISMA statement. Ann. Intern. Med..

[18] PROSPERO Registered Study Protocol. http://www.crd.york.ac.uk/PROSPERO/display_record.asp?ID=CRD42014013070#.VAihrWSSxBA.

[19] World Health Organization. Institutional Repository of Information Sharing.

[20] Crowe M. (2013). Crowe Critical Appraisal Tool (CCAT) User Guide.

[21] Jackson R., Ameratunga S., Broad J., Connor J., Lethaby A., Robb G., Wells S., Glasziou P., Heneghan C. (2006). The GATE frame: Critical appraisal with pictures. Evid. Based Nurs..

[22] World Health Organization (2005). Monitoring and Epidemiological Assessment of the Programme to Eliminate Lymphatic Filariasis at Implementation Unit Level.

[23] DerSimonian R., Laird N. (1986). Meta-analysis in clinical trials. Controll. Clin. Trials.

[24] Higgins J., Thompson S.G., Deeks J.J., Altman D.G. (2003). Measuring inconsistency in meta-analyses. BMJ.

[25] Ahmad A.F., Ngui R., Muhammad Aidil R., Lim Y.A., Rohela M. (2014). Current status of parasitic infections among Pangkor Island community in Peninsular Malaysia. Trop. Biomed..

[26] Bhumiratana A., Koyadun S., Srisuphanunt M., Satitvipawee P., Limpairojn N., Gaewchaiyo G. (2005). Border and imported bancroftian filariases: Baseline seroprevalence in sentinel populations exposed to infections with *Wuchereria bancrofti* and concomitant HIV at the start of diethylcarbamazine mass treatment in Thailand. Southeast Asian J. Trop. Med. Public Health.

[27] Bhumiratana A., Koyadun S., Suvannadabba S., Karnjanopas K., Rojanapremsuk J., Buddhirakkul P., Tantiwattanasup W. (1999). Field trial of the ICT filariasis for diagnosis of *Wuchereria bancrofti* infections in an endemic population of Thailand. Southeast Asian J. Trop. Med. Public Health.

[28] Bhumiratana A., Siriaut C., Koyadun S., Satitvipawee P. (2004). Evaluation of a single oral dose of diethylcarbamazine 300 mg as provocative test and simultaneous treatment in Myanmar migrant workers with *Wuchereria bancrofti* infection in Thailand. Southeast Asian J. Trop. Med. Public Health.

[29] Bhumiratana A., Wattanakull B., Koyadun S., Suvannadabba S., Rojanapremsuk J., Tantiwattanasup W. (2002). Relationship between male hydrocele and infection prevalences in clustered communities with uncertain transmission of *Wuchereria bancrofti* on the Thailand-Myanmar border. Southeast Asian J. Trop. Med. Public Health.

[30] Chansiri K., Phantana S. (2002). A polymerase chain reaction assay for the survey of bancroftian filariasis. Southeast Asian J. Trop. Med. Public Health.

[31] Cox-Singh J., Pomrehn A.S., Rahman H.A., Zakaria R., Miller A.O., Singh B. (1999). Simple blood-spot sampling with nested polymerase chain reaction detection for epidemiology studies on Brugia malayi. Int. J. Parasitol..

[32] Dutta P., Gogoi B.K., Chelleng P.K., Bhattacharyya D.R., Khan S.A., Goswami B.K., Mahanta J. (1995). Filariasis in the labour population of a tea estate in Upper Assam. Indian J. Med. Res..

[33] Hafiz I., Graves P., Haq R., Flora M.S., Kelly-Hope L.A. (2015). Clinical case estimates of lymphatic filariasis in an endemic district of Bangladesh after a decade of mass drug administration. Trans. R. Soc. Trop. Med. Hyg..

[34] Hakim S.L., Vythilingam I., Marzukhi M.I., Mak J.W. (1995). Single-dose diethylcarbamazine in the control of periodic brugian filariasis in Peninsular Malaysia. Trans. R. Soc. Trop. Med. Hyg..

[35] Jamail M., Andrew K., Junaidi D., Krishnan A.K., Faizal M., Rahmah N. (2005). Field validation of sensitivity and specificity of rapid test for detection of Brugia malayi infection. Trop. Med. Int. Health.

[36] Jiraamonnimit C., Wongkamchai S., Boitano J., Nochot H., Loymek S., Chujun S., Yodmek S. (2009). A cohort study on anti-filarial IgG4 and its assessment in good and uncertain MDA-compliant subjects in brugian filariasis endemic areas in southern Thailand. J. Helminthol..

[37] Khan A.M., Dutta P., Khan S.A., Baruah N.K., Sarma C.K., Mahanta J. (1999). Prevalence of bancroftian filariasis in a foot-hill tea garden of upper Assam. J. Commun. Dis..

[38] Khan A.M., Dutta P., Khan S.A., Baruah N.K., Sharma C.K., Mahanta J. (1999). Bancroftian filariasis in a weaving community of lower Assam. J. Commun. Dis..

[39] Khan A.M., Dutta P., Khan S.A., Mahanta J. (2004). A focus of lymphatic filariasis in a tea garden worker community of central Assam. J. Environ. Biol..

[40] Khan A.M., Dutta P., Khan S.A., Mohapatra P.K., Baruah N.K., Sharma C.K., Mahanta A.J. (1999). Lymphatic filariasis in two distinct communities of upper Assam. J. Commun. Dis..

[41] Khan A.M., Dutta P., Sarmah C.K., Baruah N.K., Das S., Pathak A.K., Sarmah P., Hussain M.E., Mahanta J. (2015). Prevalence of lymphatic filariasis in a tea garden worker population of Dibrugarh (Assam), India after six rounds of mass drug administration. J. Vector Borne Dis..

[42] Koyadun S., Bhumiratana A. (2005). Surveillance of imported bancroftian filariasis after two-year multiple-dose diethylcarbamazine treatment. Southeast Asian J. Trop. Med. Public Health.

[43] Koyadun S., Bhumiratana A., Prikchu P. (2003). *Wuchereria bancrofti* antigenemia clearance among Myanmar migrants after biannual mass treatments with diethylcarbamazine, 300 mg oral-dose FILADEC tablet, in Southern Thailand. Southeast Asian J. Trop. Med. Public Health.

[44] Krairittichai U., Pungprakiet D., Boonthongtho K., Arsayot K. (2012). Prevalence of infectious diseases of immigrant workers receiving health examinations at Rajavithi Hospital. J. Med. Assoc. Thail..

[45] Leang R., Socheat D., Bin B., Bunkea T., Odermatt P. (2004). Assessment of disease and infection of lymphatic filariasis in Northeastern Cambodia. Trop. Med. Int. Health.

[46] Lim B.H., Rahmah N., Afifi S.A., Ramli A., Mehdi R. (2001). Comparison of Brugia-ELISA and thick blood smear examination in a prevalence study of brugian filariasis in Setiu, Terengganu, Malaysia. Med. J. Malays..

[47] Medhi G.K., Hazarika N.C., Shah B., Mahanta J. (2006). Study of health problems and nutritional status of tea garden population of Assam. Indian J. Med. Sci..

[48] Nuchprayoon S., Porksakorn C., Junpee A., Sanprasert V., Poovorawan Y. (2003). Comparative assessment of an Og4C3 ELISA and an ICT filariasis test: A study of Myanmar migrants in Thailand. Asian Pac. J. Allergy Immunol..

[49] Nuchprayoon S., Sanprasert V., Porksakorn C., Nuchprayoon I. (2003). Prevalence of bancroftian filariasis on the Thai-Myanmar border. Asian Pac. J. Allergy Immunol..

[50] Nuchprayoon S., Yentakam S., Sangprakarn S., Junpee A. (2001). Endemic bancroftian filariasis in Thailand: detection by Og4C3 antigen capture ELISA and the polymerase chain reaction. J. Med. Assoc. Thail..

[51] Prakash A., Mohapatra P.K., Das H.K., Sharma R.K., Mahanta J. (1998). Bancroftian filariasis in Namrup tea estate, district Dibrugarh, Assam. Indian J. Public Health.

[52] Priest J.W., Jenks M.H., Moss D.M., Mao B., Buth S., Wannemuehler K., Soeung S.C., Lucchi N.W., Udhayakumar V., Gregory C.J. (2016). Integration of multiplex bead assays for parasitic diseases into a national, population-based serosurvey of women 15–39 years of age in Cambodia. PLoS Negl. Trop. Dis..

[53] Rahmah N., Lim B.H., Azian H., Ramelah T.S., Rohana A.R. (2003). Short communication: Use of a recombinant antigen-based ELISA to determine prevalence of brugian filariasis among Malaysian schoolchildren near Pasir Mas, Kelantan-Thailand border. Trop. Med. Int. Health.

[54] Rahmah N., Nurulhasanah O., Norhayati S., Zulkarnain I., Norizan M. (2010). Comparison of conventional versus real-time PCR detection of *Brugia malayi* DNA from dried blood spots from school children in a low endemic area. Trop. Biomed..

[55] Saha A.K., Mohanta M.K. (2011). Bancroftian elephantiasis in Nilphamari, Bangladesh. Mymensingh Med. J. MMJ.

[56] Samad M.S., Itoh M., Moji K., Hossain M., Mondal D., Alam M.S., Kimura E. (2013). Enzyme-linked immunosorbent assay for the diagnosis of *Wuchereria bancrofti* infection using urine samples and its application in Bangladesh. J. Parasitol. Res..

[57] Satimai W., Jiraamonnimit C., Thammapalo S., Choochote W., Luenee P., Boitano J.J., Wongkamchai S. (2011). The impact of a national program to eliminate lymphatic filariasis in selected Myanmar immigrant communities in Bangkok and Ranong Province, Thailand. Southeast Asian J. Trop. Med. Public Health.

[58] Swaddiwudhipong W., Tatip Y., Meethong M., Preecha P., Kobasa T. (1996). Potential transmission of bancroftian filariasis in urban Thailand. Southeast Asian J. Trop. Med. Public Health.

[59] Triteeraprapab S., Karnjanopas K., Porksakorn C., Sai-Ngam A., Yentakam S., Loymak S. (2001). Lymphatic filariasis caused by *Brugia malayi* in an endemic area of Narathiwat Province, southern of Thailand. J. Med. Assoc. Thail..

[60] Triteeraprapab S., Nuchprayoon I., Porksakorn C., Poovorawan Y., Scott A.L. (2001). High prevalence of *Wuchereria bancrofti* infection among Myanmar migrants in Thailand. Ann. Trop. Med. Parasitol..

[61] Triteeraprapab S., Songtrus J. (1999). High prevalence of bancroftian filariasis in Myanmar-migrant workers: A study in Mae Sot district, Tak province, Thailand. J. Med. Assoc. Thail..

[62] Wan Omar A., Sulaiman O., Yusof S., Ismail G., Fatmah M.S., Rahmah N., Khairul A.A. (2001). Epidemiological screening of lymphatic filariasis among immigrants using dipstick colloidal dye immunoassay. Malays. J. Med. Sci..

[63] World Health Organization: Regional Office for South-East Asia (2011). Elimination of Lymphatic Filariasis in the South-East Asia Region: Report of the Eighth Meeting of National National Programme Managers.

[64] World Health Organization: Regional Office for South-East Asia (2006). Elimination of Lymphatic Filariasis in the South-East Asia Region: Report of the Fifth Meeting of National National Programme Managers.

[65] United States Agency for International Development (2015). FHI360/USAID. End Neglected Tropical Diseases in Asia: Final Report.

[66] World Health Organization: Regional Office for the Western Pacific (2009). First Mekong-Plus Programme Managers Workshop on Lymphatic Filariasis and Other Helminthiasis, Phnom Penh, Cambodia, 23–26 March 2009.

[67] World Health Organization: Regional Office for the Western Pacific (2010). Malaysia: Country Cooperation Strategy 2009–2013.

[68] Myanmar Ministry of Health (2005). Myanmar National Programme to Eliminate Lymphatic Filariasis: Annual Report (2005).

[69] Myanmar Ministry of Health (2011). Myanmar National Programme to Eliminate Lymphatic Filariasis: Annual Report (2011).

[70] Myanmar Ministry of Health (2012). Myanmar National Programme to Eliminate Lymphatic Filariasis: Annual Report (2012).

[71] World Health Organization: Regional Office for South-East Asia (2004). Regional Stategic Plan. for Elimination of Lymphatic Filariasis (2004–2007).

[72] World Health Organization: Regional Office for the Western Pacific (2013). Report of the Thirteenth Meeting of the Western Pacific Regional Programme Review Group on Neglected Tropical Diseases.

[73] World Health Organization (1998). UNDP-World Bank-WHO Special Programme for Research and Training in Tropical Diseases WHO-UNICEF Joint Programme for Health Mapping World Health Organization: Division of Tropical Diseases. Research on Rapid Geographical Assessment of Bancroftian Filariasis.

[74] World Health Organization: Regional Office for South-East Asia (2011). South-East Asia Regional Programme Review Group of Elimination of Lymphatic Filariasis: Report of the Eight Meeting.

[75] World Health Organization: Regional Office for South-East Asia (2005). South.-East. Asia Regional Programme Review Group of Elimination of Lymphatic Filariasis: Report of the First Meeting.

[76] World Health Organization: Regional Office for South-East Asia (2012). South.-East. Asia Regional Programme Review Group of Elimination of Lymphatic Filariasis: Report of the Ninth Meeting.

[77] World Health Organization: Regional Office for South-East Asia (2010). South.-East. Asia Regional Programme Review Group of Elimination of Lymphatic Filariasis: Report of the Seventh Meeting.

[78] World Health Organization: Regional Office for South-East Asia (2013). South.-East. Asia Regional Programme Review Group of Elimination of Lymphatic Filariasis: Report of the Tenth Meeting.

[79] World Health Organization: Regional Office for the Western Pacific (2014). Report of the Fourteenth Meeting of the Western Pacific Regional Programme Review Group on Neglected Tropical Diseases.

[80] World Health Organization (2016). Strengthening the Assessment of Lymphatic Filariasis Transmission and Documenting the Achievement of Elimination.

[81] Cambodia Ministry of Planning (2002). General Population Census of Cambodia 1998: Final Results.

[82] Beng T.S., Ahmad R., Hisam R.S.R., Heng S.K., Leaburi J., Ismail Z., Sulaiman L.H., Soyoti R.F.H.M., Lim L.H. (2016). Molecular xenomonitoring of filarial infection in Malaysian mosquitoes under the National Program for Elimination of Lymphatic Filariasis. Southeast Asian J. Trop. Med. Public Health.

[83] Mathieu E., Amann J., Eigege A., Richards F., Sodahlon Y. (2008). Collecting baseline information for national morbidity alleviation programs: Different methods to estimate lymphatic filariasis morbidity prevalence. Am. J. Trop. Med. Hyg..

[84] Triteeraprapab S., Kanjanopas K., Suwannadabba S., Sangprakarn S., Poovorawan Y., Scott A.L. (2000). Transmission of the nocturnal periodic strain of *Wuchereria bancrofti* by *Culex quinquefasciatus*: Establishing the potential for urban filariasis in Thailand. Epidemiol. Infect..

[85] Bhumiratana A., Intarapuk A., Koyadun S., Maneekan P., Sorosjinda-Nunthawarasilp P. (2013). Current bancroftian filariasis elimination on Thailand-Myanmar border: Public Health Challenges toward Postgenomic MDA Evaluation. ISRN Trop. Med..

[86] Toothong T., Tipayamongkholgul M., Suwannapong N., Suvannadabba S. (2015). Evaluation of mass drug administration in the program to control imported lymphatic filariasis in Thailand. BMC Public Health.

