# Supplementary material for: Lymphatic Filariasis in Mainland Southeast Asia: A Systematic Review and Meta-Analysis of Prevalence and Disease Burden
Source: Trop Med Infect Dis. 2017 Jul 27;2(3):32. doi: 10.3390/tropicalmed2030032 (PMC6082107; doi:10.3390/tropicalmed2030032)
Supplement: Supplementary file 1 [file tropicalmed-02-00032-s001.pdf]

Tropical Medicine and Infectious Disease

MDPI

Supplementary Materials: Lymphatic Filariasis in Mainland Southeast Asia: A Systematic Review and Meta-Analysis of Prevalence and Disease Burden

Benjamin F. R. Dickson, Patricia M. Graves and William J. McBride

Table S1. Quality Assessment of Included Peer-Reviewed Journal Articles.

Study *Author (publication year) [study year] [reference]*	Sampling			Data Collection Adequate		Total Score
	Sample Size	Location	Participants	Infection Assessment	Morbidity Assessment	
Bangladesh						
Hafiz et al. (2015) [2011] [33]	+++	Y	Y	Y	Y	10/10
Saha et al. (2011) [NS[d]] [55]	+++	?	Y	-	Y	7/8
Samad et al. (2013) [NS] [56]	++	?	?	Y	-	5/8
Cambodia						
Leang et al. (2004) [2000–2001] [45]	+++	Y	Y	Y	Y	10/10
Priest et al. (2016) [2012] [52]	+++	Y	Y	Y	-	8/8
India						
Dutta et al. (1995) [1992] [32]	+++	?	Y	Y	Y	9/10
Khan et al. (1999) [NS] Study 1 [37]	++	Y	Y	Y	?	8/10
Khan et al. (1999) [NS] Study 2 [40]	+++	Y	Y	Y	?	9/10
Khan et al. (1999) [NS] Study 3 [38]	++	?	?	Y	-	5/8
Khan et al. (2004) [NS] [39]	++	Y	Y	Y	Y	9/10
Khan et al. (2015) [2012-2013] [41]	++	?	?	Y	Y	7/10
Medhi et al. (2006) [2002–2003][47]	+++/+[a]	Y	Y	Y	Y	9/10
Prakash et al. (1998) [1994] [51]	+++	Y	Y	Y	Y	10/10
Malaysia						
Ahmad et al. (2014) [NS] [25]	+	?	?	Y	-	4/8
Cox-Singh et al. (1999) [NS] [31]	+	?	Y	Y	-	5/8
Hakim et al. (1995) [1992] [34]	++	N	Y	Y	-	5/8
Jamail et al. (2005) [2001–2002] [35]	+++	N	?	Y	-	5/8
Lim et al (2001) [NS] [46]	+++	N	?	Y	-	5/8
Rahmah et al. (2003) [NS] [53]	+++	Y	Y	Y	-	8/8
Rahmah et al. (2010) [?] [54]	++	N	?	Y	-	4/8
Wan Omar et al. (2001) [NS] [62]	++	?	N[b]	Y	Y	6/10
Thailand						
Bhumiratana et al. (2004) [2002] [28]	++/+[a]	?	?	Y	-	4.5/8
Bhumiratana et al. (2005) [1998–2001] [26]	++	?	?	Y	-	5/8
Bhumiratana et al. (1999) [1998] [27]	+	?	?	Y	Y	6/10
Bhumiratana et al (2002) [1999][29]	+[c]	N	Y	Y	Y	6/10
Chansiri et al. (2002)[1997–2001][30]	+++	?	?	N	-	4/8
Jiraamonnimit et al. (2009) [2005–2006] [36]	++	?	Y	Y	?	7/10
Koyadun et al. (2003) [2001–2002] [43]	+++	N	?	Y	-	5/8
Koyadun et al. (2005) [2003] [42]	++	?	Y	Y	-	6/10
Krairittichai et al. (2012) [2010] [44]	+++	Y	Y	?	-	7/8
Nuchprayoon et al. (2003) [NS] Study 1 [48]	++/+[a]	?	N[b]	Y	Y	5.5/10
Nuchprayoon et al. (2003) [NS] Study 2 [49]	++/+[a]	?	?	Y	-	4.5/8
Nuchprayoon et al. (2001) [NS] [50]	+	?	?	Y	-	4/8
Satimai et al. (2011) [NS] [57]	+++	?	?	Y	-	6/8
Swaddiwudhipong et al.(1996) [1995] [58]	+++	?	?	Y	?	7/10
Triteeraprapab et al. (1999) [NS][61]	++	?	?	Y	-	5/8

Triteeraprapab et al. (2001) [NS] Study 1 [59]	+++	?	?	Y	-	6/8
Triteeraprapab et al. (2001) [1999] Study 2 [60]	++	N	Y	Y	-	5/8

Y= Sampling/Assessment adequate (2 points). ? = Not clear/not stated (1 point). N= Sampling/Assessment not adequate (0 points).

+++ = good (>1000) (2 points), ++ = adequate (300-1000) (1 point), + = poor (<300) (0 points)

[a]Total sample size/subset sample size , [b]Excluded those with DEC treatment in the last 12 months, [c]Poor size, however includes the whole village, [d]Not stated

Table S2. Infection and Morbidity Prevalence by Country and Region in Peer-Reviewed Journals;

Province/State	[Reference], Study Year	Infection Prevalence (%)							Morbidity Prevalence (%)		MDA Status[k]
		Wb	Bm	Total Mf[a]	ICT[b]	Og4C3[c]	IgG4[d]	Spp.[e]	Lymph[e]	Hyd[f]	
Bangladesh											
Nilphamari	[55], 2011							-	0.45		Mid
	[33], 2011	1.13	0	1.13	1.70			Wb	2.66	4.16	Mid
Panchargarh	[56], 2013*				0.31		2.19	Wb			Post
Cambodia											
Mondulkiri	[45], 2001	0	0	0	0			-	0	0	Pre
Preah Vihear	[45], 2001	0	0	0	0.22			Wb	0.44	2.51	Pre
Rattanakiri	[45], 2001	0.32	0.81	1.13	1.94			Wb, Bm	1.00	3.95	Pre
Strung Treng	[45], 2001	0.09	0	0.09	0.38			Wb	0.38	1.91	Pre
North Region	[52], 2012[#]						6.60	Wb (Bm)			Post
West Region	[52], 2012[#]						1.57	Wb (Bm)			Post
South-East Region	[52], 2012[#]						1.19	Wb (Bm)			Post
South-West Region	[52], 2012[#]						1.65	Wb (Bm)			Post
Phnom Penh	[52], 2012[#]						1.49	Wb (Bm)			Post
India											
Assam	[32], 1992[j]	8.31	0	8.31				Wb	0.48[h]		Pre
	[51], 1994[j]	6.70	0	6.70				Wb	0.72	4.25	Pre
	[37], 1999[j]	0.61	0	0.61				Wb	0.12	1.01	Pre
	[40], 1999	0.45	0	0.45				Wb	0[h]		Pre
	[40], 1999[j]	10.27	0	10.27				Wb	1.41[h]		Pre
	[38], 1999	1.79	0	1.79				Wb			Pre
	[47], 2003[j]	8.66	0	8.66				Wb	0.57[h]		Pre
	[39], 2004[j]	4.73	0	4.73				Wb	0.61	7.08	Pre
	[41], 2013[j]	7.41	0	7.41				Wb	0.52	8.96	Mid
Malaysia											
Kelantan	[53], 2003*						0.35	Bm			Pre
Perak	[34], 1992	0	23.85	23.85				Bm			Pre
	[25], 2014	0	0	0				-			Mid
Perlis	[54], 2010*						2.16	Bm			Pre[&]
Sabah	[31], 1999	0	20.69	20.69				Bm			Pre
Trengganu	[46], 2001	0	0.26	0.26			2.47	Bm			Pre
	[62], 2001[i]	5.24	8.10	13.33				Bm, Wb	0		Pre
Sarawak	[35], 2002	0	0.90	0.90			9.35	Bm			Pre
Thailand											

Bangkok	[44], 2010 (Laos)			0				-			Post
	[44], 2010 (Myanmar)			0.01				NS			Post
	[44], 2010 (Cambodia)			0				-			Post
	[57], 2011[i]				1.14		4.79	Wb			Post
	[57], 2011				0		0	-			Post
Southern Thai Peninsula											
Nakhon Srithamarat	[36], 2006	0	0	0			8.00	Bm			Mid
Narathiwatt	[59], 2001	0	1.38	1.38				Bm			Pre
	[36], 2006						23.67	Bm			Mid
Phang-Nga	[43], 2002[i]				9.52			Wb			Pre
	[43], 2002				0			Wb			Pre
	[28], 2004[i]	0.23	0	0.23	7.00	9.00		Wb			Mid
Ranong	[42], 2003[i]	0.82	0	0.82				Wb			Mid
	[42], 2003[i]	1.19	0	1.19				Wb			Mid
	[57], 2011[i]				0.20		2.73	Wb			Post
Suratthani	[36], 2006	0	2.00	2.00			19.00	Bm			Mid
Narathiwatt, Nakorn Srithammarat & Suratthani Provinces	[36], 2006								1.20		Mid
Thai-Myanmar Border											
Suphanburi	[28], 2002[i]	0.59	0	0.59	3.00	4.00		Wb			Mid
Tak	[58], 1995[i]	2.49	0	2.49				Wb	0.06[h]		Pre
	[27], 1998	5.78	0	5.78	20.00			Wb		8.15	Pre
	[29], 1999	1.01	0	1.01	13.13			Wb		0	Pre
	[29], 1999[i]	5.83	0	5.83	32.50			Wb		16.13	Pre
	[61], 1999[i]	4.43	0	4.43				Wb			Pre
	[60], 1999[i]	7.82	0	7.82		10.24	42.32	Wb			Pre
	[26], 2001[i]	3.17	0	3.17	13.57	23.98		Wb			Pre
	[26], 2001	6.13	0	6.13	26.42	36.79		Wb			Pre
	[50], 2001	10.20	0	10.20		22.45		Wb			Pre
	[28], 2002[i]	0	0	0	2.30	4.60		Wb			Mid
	[48], 2003[i]	3.26	0	3.26	12.73	19.49		Wb		8.62	Pre
	[49], 2003	5.54	0	5.54		21.89	53.93	Wb			Pre

[a] Microfilaraemia via TBS. [b] Immunochromatographic card test (ICT). [c] Og4C3 ELISA. [d] Anti-Bm14 or Anti-BmR1 Antibodies. [e] *W. bancrofti* (Wb), *B. malayi* (Bm), [f] Lymphoedema. [g] Hydrocoele. [h] Only combined morbidity reported. [I] Migrant population, [j] Tea estate population, [K] Status of National Elimination Programme: Pre-MDA (Pre), Mid-MDA (Mid), Post-MDA (Post), [&] MDA not conducted in this area, * Only sampled children, [#] Only sampled women 15–39

Table S3. Infection and Morbidity Prevalence by Country and Region in Grey Literature;

Province	[Reference], Publication Year	Infection Prevalence (%)			Morbidity Prevalence (%)		MDA Status[f]
		Mf[b]	ICT[c]	Spp.	Lymph.[d]	Hyd.[e]	
Bangladesh							
National Prevalence Data	[5], 2004	1.00	0.03	Wb			Mid
	[5], 2005	0.30					Mid
	[5], 2006	0.60					Mid
	[5], 2007	0.31					Mid
	[5], 2008	0.62					Mid
	[5], 2009	0.17					Mid
	[5], 2010	0					Mid
	[5], 2011				0.10	0.27	Mid

Cambodia							
Stung Treng Province	[66], 2001	0.38[a]		Wb, Bm			Pre
	[65,66], 2005	0.80[a]					Pre
	[66], 2006	0[a]					Mid
	[65,66], 2007	0					Mid
	[65,66], 2008	0					Mid
	[65], 2009	0					Mid
	[65], 2010*		0.30				Post
	[65], 2013*		0				Post
	[65], 2015*		0				Post
Rattankiri Province	[65,66], 2001	2.75[a]					Pre
	[66], 2005	1.80[a]					Pre
	[66], 2006	0[a]					Mid
	[65,66], 2007	0					Mid
	[65,66], 2008	0					Mid
	[65], 2009	0					Mid
	[65], 2010*		0.40				Post
	[65], 2013*		0				Post
	[65], 2015*		0				Post
Siem Reap Province	[66], 2004		0.67				Pre
	[66], 2005	0[a]					Mid
	[66], 2006	0[a]					Mid
	[65,66], 2007	0					Mid
	[65,66], 2008	0					Mid
	[65], 2009	0					Mid
	[65], 2010*		0.30				Post
	[65], 2013*		0				Post
	[65], 2015*		0				Post
Preah Vihea Province	[66], 2001	0.22[a]					Pre
	[66], 2004		0.40				Pre
	[66], 2005	0[a]					Mid
	[66], 2006	0[a]					Mid
	[65,66], 2007	0					Mid
	[66], 2008	0					Mid
	[65], 2009	0					Mid
	[65], 2010*		0.20				Post
	[65], 2013*		0				Post
	[65], 2015*		0				Post
India							
National Prevalence Data	[5], 2004	1.24		Wb, Bm			Pre
	[5], 2005	1.02					Mid
	[5], 2006	0.98					Mid
	[5], 2007	0.64					Mid
	[5], 2008	0.53					Mid
	[5], 2009	0.65			0.01	0.01	Mid
	[5], 2010	0.41			0.13		Post
	[5], 2011	0.35			0.14	0.07	Post
Laos							
National Prevalence Data	[65,66], 2002 – 2006	0		Wb			Pre
	[64], 2007	0.25					Pre
Attapue	[65], 2007	0.77					Pre
	[65], 2009		11.40				Pre
	[65], 2014*		0.75				Mid
Sekong	[65], 2014		1.20				Mid
Malaysia							

Peninsular Malaysia	[66,67], 1991	0.30		Bm (Wb)			Pre
	[66,67], 1993	0.20					Pre
	[66,67], 1995	0.24					Pre
	[66,67], 1997	0.26					Pre
	[66,67], 1999	0.35					Pre
	[66,67], 2001	0.18					Pre
	[66,67], 2003	0.20					Pre
Sabah & Sarawak	[66,67], 1991	1.30					Pre
	[66,67], 1993	1.20					Pre
	[66,67], 1995	1.40					Pre
	[66,67], 1997	0.80					Pre
	[66,67], 1999	0.70					Pre
	[66,67], 2001	1.20					Pre
	[66,67], 2003	1.50					Pre
Myanmar							
Ayeyarwaddy	[73], 1997	0 – 5		Wb			Pre
Bago	[73], 1997	5 – 99					Pre
Chin	[69], 2011	1.4-8					Mid
Chin	[73], 1997	2 – 5					Pre
Kachin	[73], 1997	2 – 10					Pre
Kayah	[73], 1997	0					Pre
Kayin	[73], 1997	15 – 25					Pre
	[70], 2012	0-1					Mid
Magway	[70], 2012	0-33					Mid
	[69], 2011	0.2					Mid
	[68], 2005	0-18					Mid
	[73], 1997	5 – 99					Pre
Mandalay	[69], 2011	1.8					Mid
	[68], 2005	7.3-39					Mid
	[73], 1997	0 – 99					Pre
Mon	[73], 1997	2 – 99					Pre
Rakhine	[68], 2005	0-65					Mid
	[73], 1997	5 - 15					Pre
Sagaing	[70], 2012	0-9					Mid
	[68], 2005	0-35			0.19%	0.59%	Mid
	[73], 1997	2 – 99					Pre
Shan	[73], 1997	0 – 5					Pre
Yangon	[73], 1997	0 – 10					Pre
	[70], 2012	0-2					Mid
National Prevalence Data	[5], 2001	7.1					Pre
	[5], 2002	15.1					Pre
	[5], 2003	7.6					Mid
	[5], 2004	6.60					Mid
	[5], 2005	3.20					Mid
	[5], 2006	5.6					Mid
	[5], 2007	2.8					Mid
	[5], 2008	2.7					Mid
	[5], 2009	2.7					Mid
	[5], 2010	2.7					Mid
Thailand							
National Prevalence Data	[5], 2002	<1.0	0.08	Wb, Bm			Pre
	[5], 2003	0.77					Mid
	[5], 2004	0.15					Mid
	[5], 2005	0.16					Mid
	[5], 2006	0.04					Mid
	[5], 2007	0.06					Mid

	[5], 2008	0.03					Mid
	[5], 2009	0.05					Mid
	[5], 2010	0.09			0.12		Mid
Vietnam							
Red River Delta							
Phu Cu	[66], 2002	0.08		Bm (Wb)			Pre
	[66], 2005-2006	0					Mid
	[66], 2007-2008	0					Mid
Binh Luc	[66], 2002	0.40					Pre
	[66], 2005-2006	0					Mid
	[66], 2007-2008	0					Mid
South Central Coast							
Khanh Vinh	[66], 2002	3.64		Wb (Bm)			Pre
	[66], 2005-2006	0.62					Mid
	[66], 2007-2008	0					Mid
Dien Khanh	[66], 2002	0.27					Pre
	[66], 2005-2006	0					Mid
	[66], 2007-2008	0					Mid
Ninh Hoa	[66], 2002	3.30					Pre
	[66], 2005-2006	0.98					Mid
	[66], 2007-2008	0					Mid
Bac Ai	[66], 2002	0.15					Pre
	[66], 2005-2006	0.10					Mid
	[66], 2007-2008	0					Mid

[a] Mapping by ICT/Mf. [b] Microfilaraemia via TBS. [c] Immunochromatographic card test (ICT). [d] Lymphoedema. [e] Hydrocoele, [f] Status of National Elimination Programme: Pre-MDA (Pre), Mid-MDA (Mid), Post-MDA (Post), * Only sampled children, [%] Self-reported questionnaire.

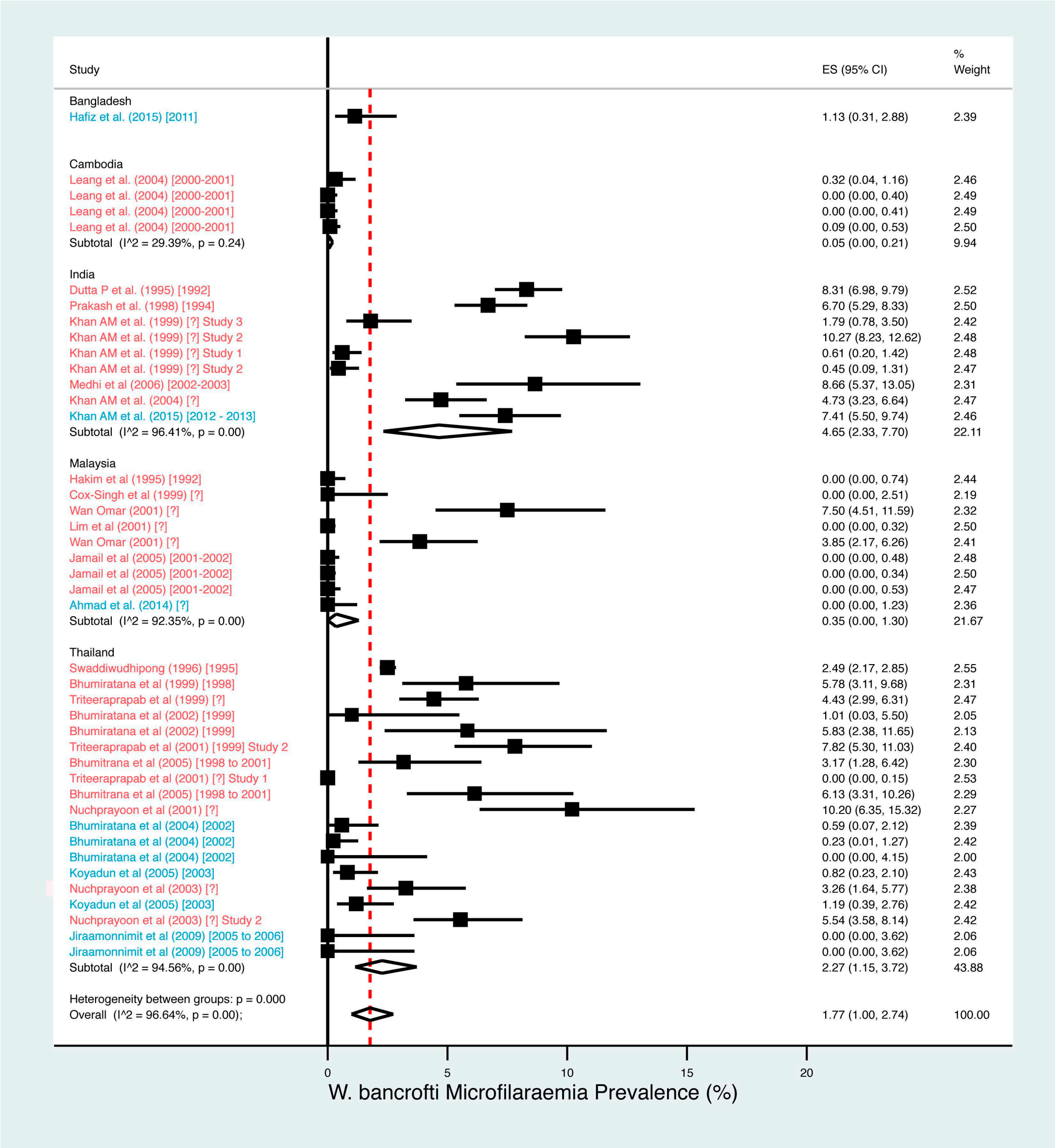

Figure S1. Percentage estimates of *W. bancrofti* microfilaraemia prevalence by country and year. ES: prevalence estimate. Red-dotted line: overall estimate. Blue diamond: sub-group estimate. Horizontal line: 95% CI. Red study: Pre-MDA, Blue study: Mid-MDA, Green study: Post-MDA.

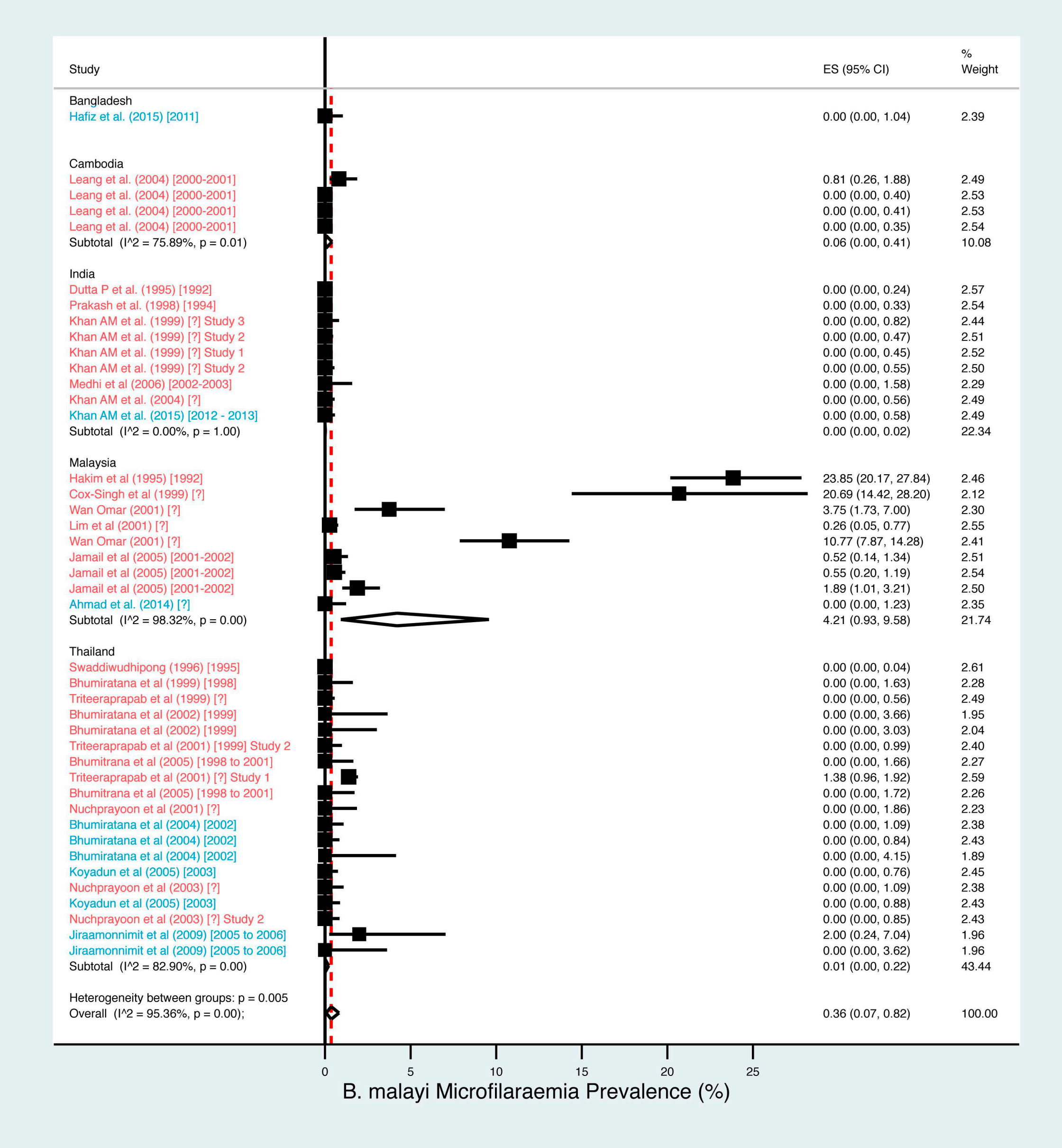

Figure S2. Percentage estimates of *B. malayi* microfilaraemia prevalence by country and year. ES: prevalence estimate. Red-dotted line: overall estimate. Blue diamond: sub-group estimate. Horizontal line: 95% CI. Red study: Pre-MDA, Blue study: Mid-MDA, Green study: Post-MDA.

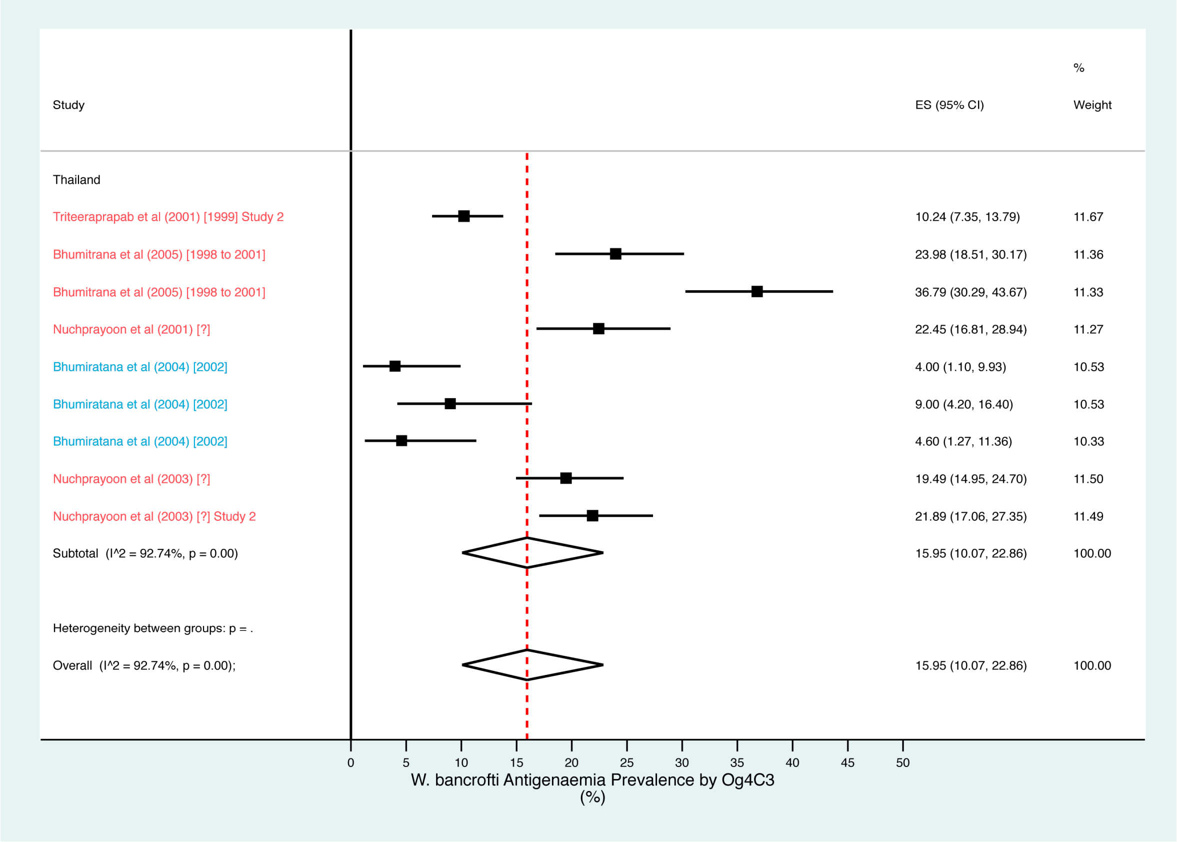

Figure S3. Percentage estimates of Og4C3 antigenaemia prevalence by country and year. ES: prevalence estimate. Red-dotted line: overall estimate. Blue diamond: sub-group estimate. Horizontal line: 95% CI. Red study: Pre-MDA, Blue study: Mid-MDA, Green study: Post-MDA.

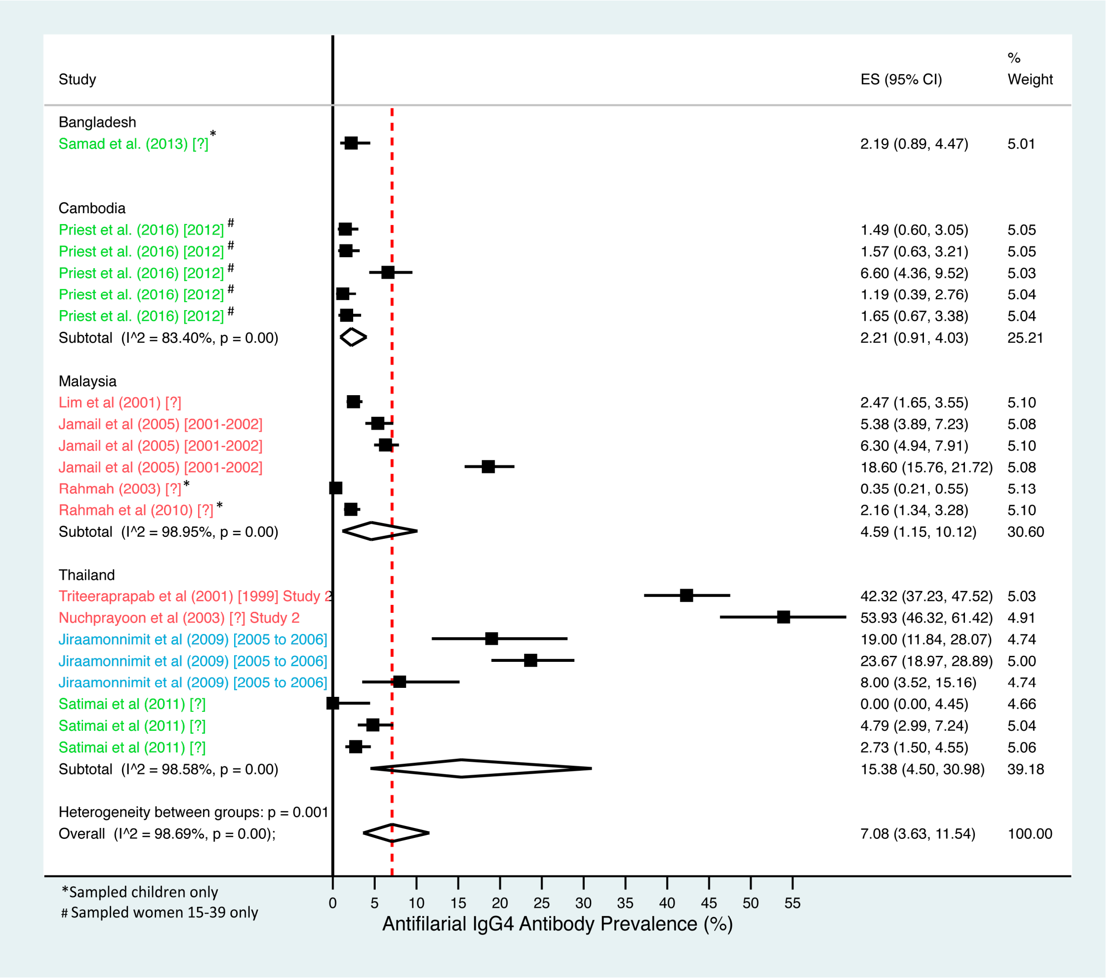

Figure S4. Percentage estimates of IgG4 antibody prevalence by country and year. ES: prevalence estimate. Red-dotted line: overall estimate. Blue diamond: sub-group estimate. Horizontal line: 95% CI. Red study: Pre-MDA, Blue study: Mid-MDA, Green study: Post-MDA.

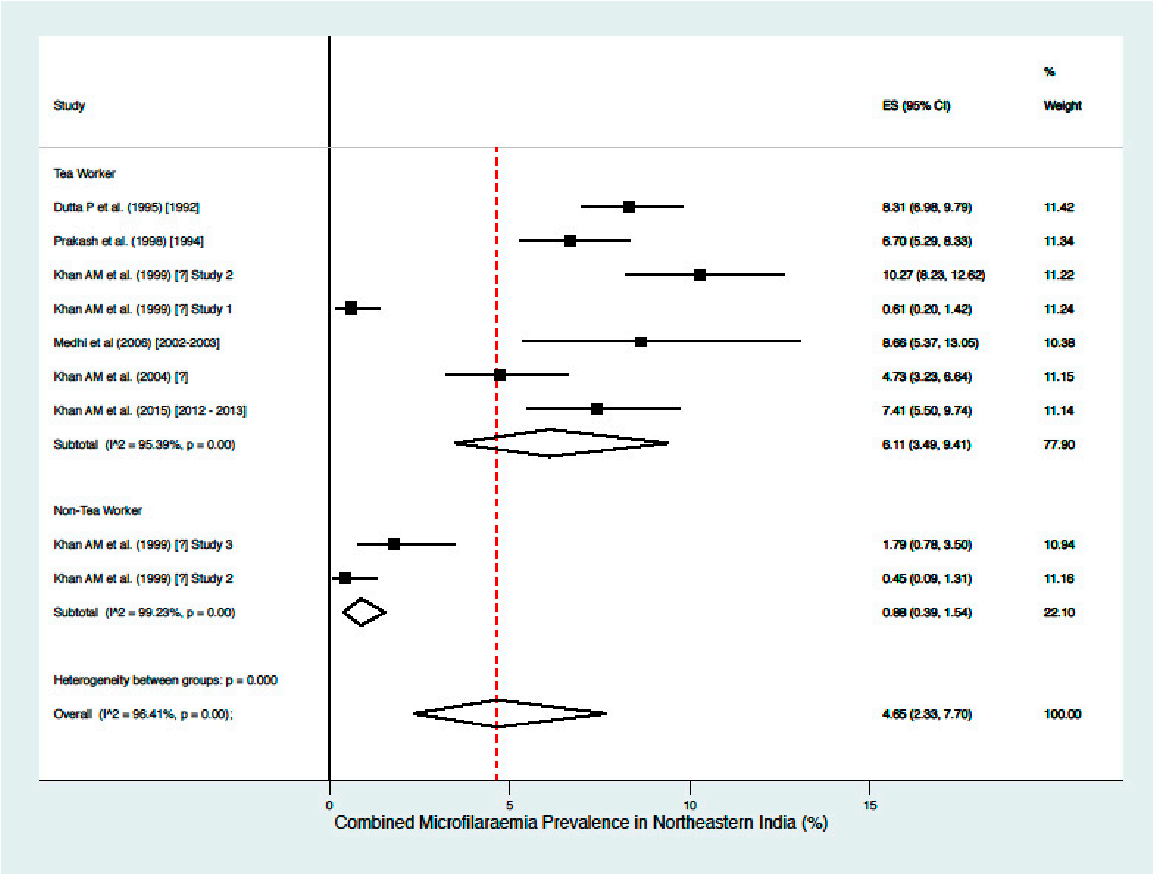

Figure S5. Percentage estimates of combined microfilaraemia prevalence in North East India. ES: prevalence estimate. Red-dotted line: overall estimate. Blue diamond: sub-group estimate. Horizontal line: 95% CI.

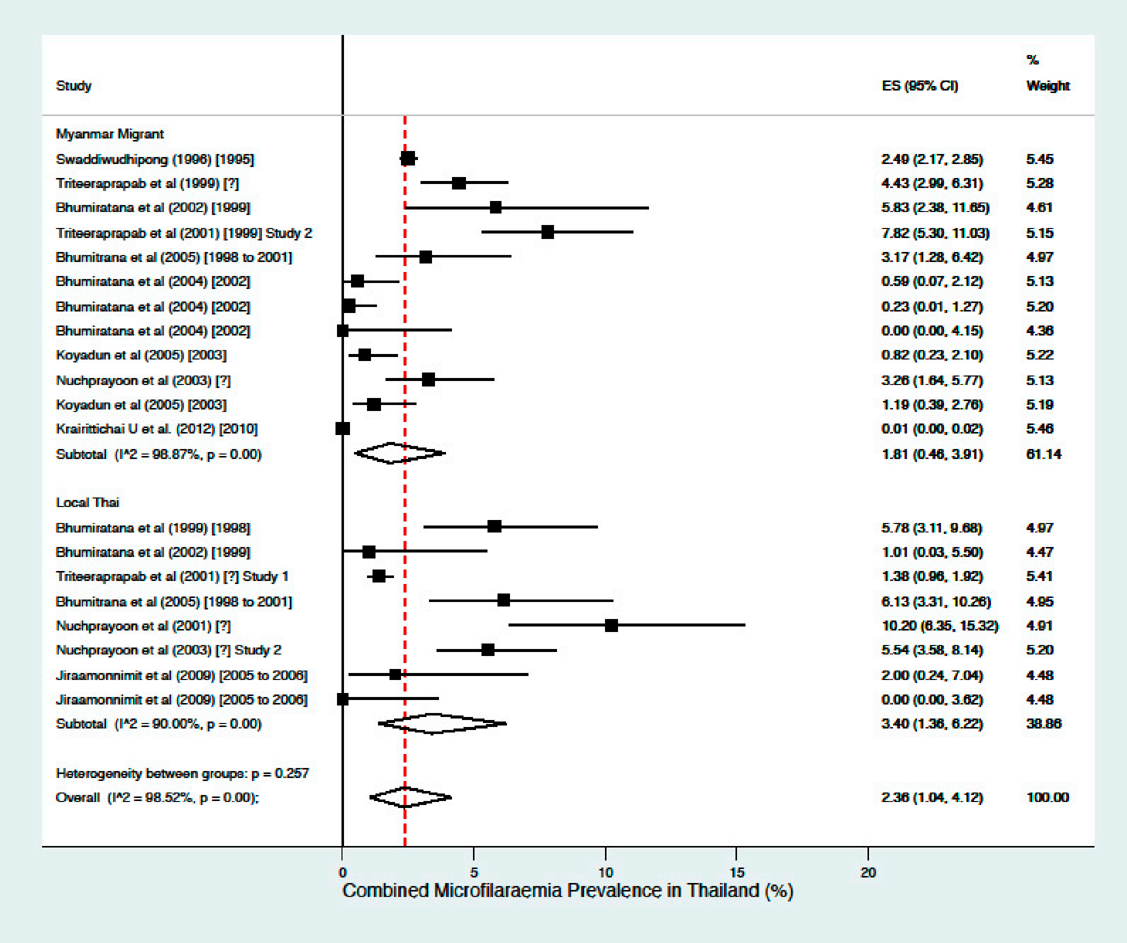

Figure S6. Percentage estimates of combined microfilaraemia prevalence in Thailand. ES: prevalence estimate. Red-dotted line: overall estimate. Blue diamond: sub-group estimate. Horizontal line: 95% CI.

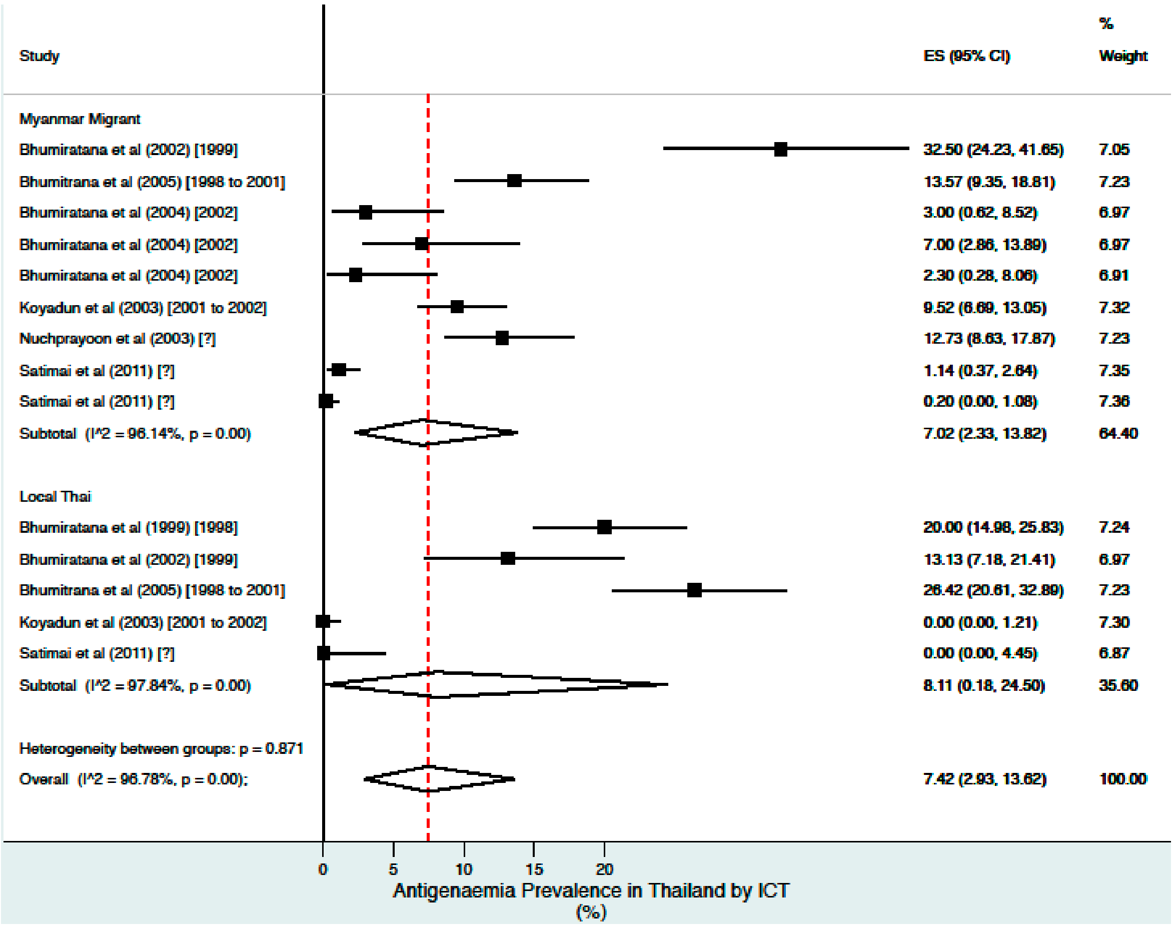

Figure S7. Percentage estimates of ICT antigenaemia prevalence in Thailand. ES: prevalence estimate. Red-dotted line: overall estimate. Blue diamond: sub-group estimate. Horizontal line: 95% CI.

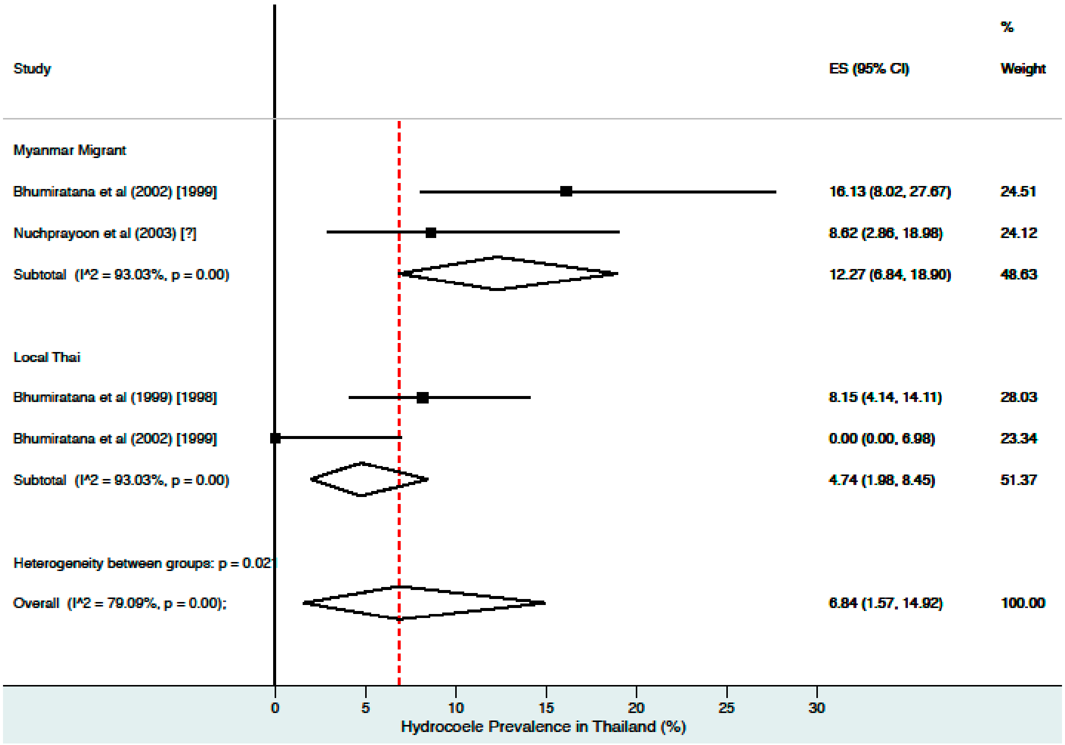

Figure S8. Percentage estimates of hydrocoele prevalence in Thailand. ES: prevalence estimate. Red-dotted line: overall estimate. Blue diamond: sub-group estimate. Horizontal line: 95% CI.

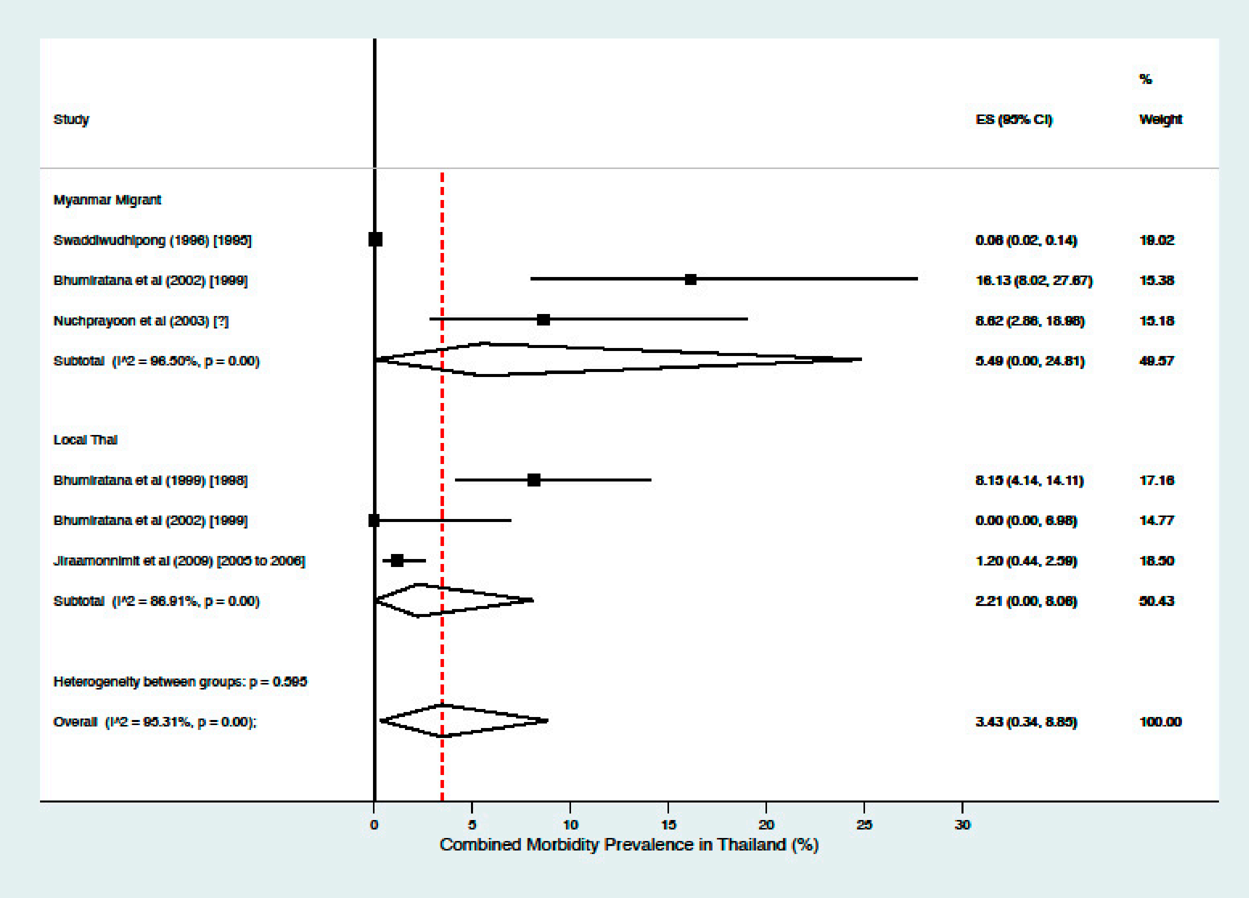

Figure S9. Percentage estimates of combined morbidity prevalence in Thailand. ES: prevalence estimate. Red-dotted line: overall estimate. Blue diamond: sub-group estimate. Horizontal line: 95% CI.

PRISMA 2009 Checklist

Section/topic	#	Checklist item	Reported on page #
**TITLE**			
Title	1	Identify the report as a systematic review, meta-analysis, or both.	1
**ABSTRACT**			
Structured summary	2	Provide a structured summary including, as applicable: background; objectives; data sources; study eligibility criteria, participants, and interventions; study appraisal and synthesis methods; results; limitations; conclusions and implications of key findings; systematic review registration number.	1
**INTRODUCTION**			
Rationale	3	Describe the rationale for the review in the context of what is already known.	2
Objectives	4	Provide an explicit statement of questions being addressed with reference to participants, interventions, comparisons, outcomes, and study design (PICOS).	2
**METHODS**			
Protocol and registration	5	Indicate if a review protocol exists, if and where it can be accessed (e.g., Web address), and, if available, provide registration information including registration number.	3
Eligibility criteria	6	Specify study characteristics (e.g., PICOS, length of follow-up) and report characteristics (e.g., years considered, language, publication status) used as criteria for eligibility, giving rationale.	3
Information sources	7	Describe all information sources (e.g., databases with dates of coverage, contact with study authors to identify additional studies) in the search and date last searched.	3
Search	8	Present full electronic search strategy for at least one database, including any limits used, such that it could be repeated.	3
Study selection	9	State the process for selecting studies (i.e., screening, eligibility, included in systematic review, and, if applicable, included in the meta-analysis).	3
Data collection process	10	Describe method of data extraction from reports (e.g., piloted forms, independently, in duplicate) and any processes for obtaining and confirming data from investigators.	3
Data items	11	List and define all variables for which data were sought (e.g., PICOS, funding sources) and any assumptions and simplifications made.	3
Risk of bias in individual studies	12	Describe methods used for assessing risk of bias of individual studies (including specification of whether this was done at the study or outcome level), and how this information is to be used in any data synthesis.	4
Summary measures	13	State the principal summary measures (e.g., risk ratio, difference in means).	4
Synthesis of results	14	Describe the methods of handling data and combining results of studies, if done, including measures of consistency (e.g., I^2) for each meta-analysis.	4

Page 1 of 2

Figure S10. PRISMA checklist.
